# Encoding quantized fluorescence states with fractal DNA frameworks

**DOI:** 10.1038/s41467-020-16112-z

**Published:** 2020-05-04

**Authors:** Jiang Li, Jiangbing Dai, Shuoxing Jiang, Mo Xie, Tingting Zhai, Linjie Guo, Shuting Cao, Shu Xing, Zhibei Qu, Yan Zhao, Fei Wang, Yang Yang, Lei Liu, Xiaolei Zuo, Lihua Wang, Hao Yan, Chunhai Fan

**Affiliations:** 1 Bioimaging Center, Shanghai Synchrotron Radiation Facility, Zhangjiang Laboratory, Shanghai Advanced Research Institute, Chinese Academy of Sciences, 201204 Shanghai, China; 2 Division of Physical Biology, CAS Key Laboratory of Interfacial Physics and Technology, Shanghai Institute of Applied Physics, Chinese Academy of Sciences, 201800 Shanghai, China; 3 Center for Molecular Design and Biomimetics, The Biodesign Institute, School of Molecular Sciences, Arizona State University, Tempe, AZ 85287 USA; 4 School of Chemistry and Chemical Engineering, Frontiers Science Center for Transformative Molecules, Institute of Molecular Medicine, Renji Hospital, School of Medicine, Shanghai Jiao Tong University, 200024 Shanghai, China; 5 Shanghai Key Laboratory of Green Chemistry and Chemical Processes, School of Chemistry and Molecular Engineering, East China Normal University, 500 Dongchuan Road, 200241 Shanghai, China

**Keywords:** Fluorescence imaging, DNA nanostructures

## Abstract

Signal amplification in biological systems is achieved by cooperatively recruiting multiple copies of regulatory biomolecules. Nevertheless, the multiplexing capability of artificial fluorescent amplifiers is limited due to the size limit and lack of modularity. Here, we develop Cayley tree-like fractal DNA frameworks to topologically encode the fluorescence states for multiplexed detection of low-abundance targets. Taking advantage of the self-similar topology of Cayley tree, we use only 16 DNA strands to construct n-node (n = 53) structures of up to 5 megadalton. The high level of degeneracy allows encoding 36 colours with 7 nodes by site-specifically anchoring of distinct fluorophores onto a structure. The fractal topology minimises fluorescence crosstalk and allows quantitative decoding of quantized fluorescence states. We demonstrate a spectrum of rigid-yet-flexible super-multiplex structures for encoded fluorescence detection of single-molecule recognition events and multiplexed discrimination of living cells. Thus, the topological engineering approach enriches the toolbox for high-throughput cell imaging.

## Abstract (web summary)

Though DNA framework-based scaffolds for biomolecular assembly are attractive for bioimaging applications, realizing super-multiplex fluorescent amplifiers remains a challenge. Here, the authors report a topological engineering approach to designing fractal DNA frameworks for multiplexed amplifiers.

## Introduction

Recruitment of multiple copies of effector molecules represents a natural strategy for amplifying weak signals from ligand-receptor interactions in various signal pathways in vivo^1,2^. This principle has been harnessed in synthetic systems for developing signal amplifiers for biosensors, cellular imaging, and genetic engineering^3–5^. For example, by recruiting multiple copies of fused fluorescent proteins to the repeating protein-binding motifs (e.g., MS2) localised on the target RNA of interest, Singer and coworkers established a method for directly visualising single RNA molecules in living cells^6,7^. Similar strategies have been employed for the CRISPR/Cas9 imaging of low-copy and non-repeating chromatin loci^8,9^. The more recently developed SunTag^10–13^ technology utilises peptide scaffolds that can recruit up to 24 copies of effector proteins to drastically amplify fluorescent signals for chromatin and RNA translation imaging^14–17^. However, due to the lack of precise spatial arrangments of the signal molecules on the peptide scaffolds, there is either incomplete labelling or background from excessive signal molecules that limit the multiplexity of SunTags^18^. The multiplexity of fluorescence imaging is also affected by the interference between FRET pairs with overlapping spectra that generally has a practical limit of 4–5 colours^19–21^.

Structural DNA nanotechnology provides a powerful platform to construct self-assembled DNA nanostructures with near-atomic resolution and to programme the spatial organisation of molecules and nanoparticles with sub-10-nm resolution^22–30^. Specifically, the use of DNA frameworks as biocompatible scaffolds for programmable biomolecular assembly has been actively explored for various diagnostic, bioimaging and drug delivery applications^5,31–41^. However, the regular, monomeric DNA frameworks do not satisfy the requirements for developing super-multiplex fluorescent amplifiers. For example, small-sized tetrahedral DNA nanostructures (TDNs) of 5–20 nm can only accommodate a few molecules in a single structure^35,39,42^; whereas the complexity to construct large-sized DNA origamis or single-stranded-tile-based structures of >100 nm with hundreds or even thousands of distinct DNA strands makes them practically difficult to use^43–46^.

In this work, we develop Cayley tree-like fractal DNA frameworks (FDFs) to break the size limit with minimal use of DNA strands, and to improve the modularity for multiplexing. Cayley trees are acyclic graph structures with each node linked to a consistent number of neighbour nodes (i.e., coordination number). We show that the use of TDNs as nodes with 4-coordination generates rigid-yet-flexible FDFs with molecular weights up to ~5 megadalton. By exploiting the self-similar topology (node connection arrangement), we construct FDF amplifiers with only a small set of 16 DNA strands which can encode up to 36 distinct colours with minimal crosstalk. These FDFs can topologically encode quantitative fluorescence states for single-molecule recognition and multiplexed cell discrimination.

## Results

### Construction of fractal DNA frameworks

**Fig. 1:**
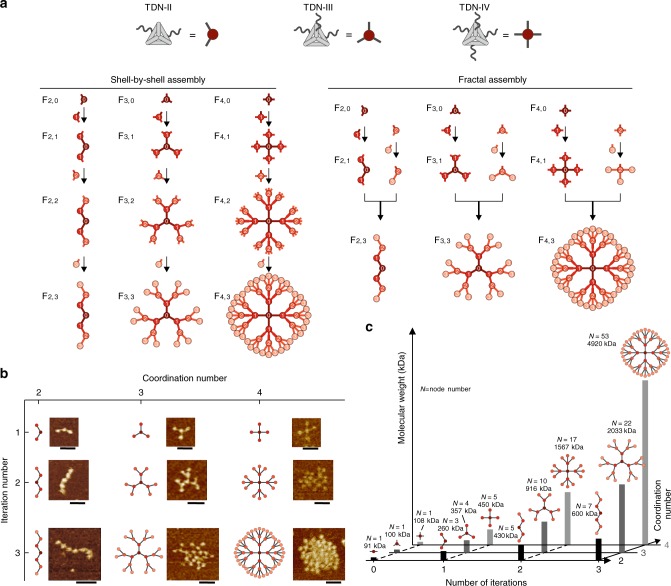
Fractal DNA frameworks (FDFs) with precise node numbers and molecular weights. **a** Schematic of FDFs’ assembly strategies. **b** Representative AFM images of the FDFs. Scale Bar, 50 nm. **c** Comparison diagram of FDFs with different node numbers (*N*) and molecular weights.

In our design, a Cayley tree-like FDF (denoted as *F*_n,i_, where *n* is the coordination number of each node, and *i* is the iteration number of the structure, shown in Fig. 1a) is assembled with tetrahedral DNA nodes (TDNs). Each TDN (edge length 20 bp, or ~6.8 nm, adapted from previous studies^35,42^, see Methods and Supplementary Table 1) is assembled from four oligonucleotides, and carries *n* (*n* = 2, 3 or 4) single-stranded overhangs for inter-node recognition (denoted as TDN-II, TDN-III or TDN-IV, respectively, shown in Supplementary Fig. 1). The root TDN (shell-0) with *n* overhangs can recognise and assemble with *n* TDNs in the exterior shell (shell-1) through hybridisation of the overhangs to form an *F*_*n*,1_ structure containing *n* + 1 nodes. The remaining overhangs of the shell-1 nodes in turn recruit additional nodes to form *F*_*n*,2_ and so forth (Supplementary Table 2). Using this shell-by-shell assembly approach (in a sequence of shell-0 + shell-1 + shell-2 + shell-3), we synthesised FDFs with 1–3 shells (including *F*_2,1_, *F*_2,2_, *F*_2,3_, *F*_3,1_, *F*_3,2_, *F*_3,3_, *F*_4,1_, *F*_4,2_ and *F*_4,3_, respectively). Alternatively, fractal assembly was used for the synthesis of 3-shell trees (*F*_2,3_, *F*_3,3_ and *F*_4,3_, shown in Fig. 1a): shell-0 nodes were first assembled with shell-1 nodes (shell-0 + shell-1) to form *F*_2,1_, *F*_3,1_ and *F*_4,1_ (same to the shell-by-shell approach); in parallel, shell-2 nodes were coupled with shell-3 nodes elsewhere to form partial assemblies (shell-2 + shell-3), which were then assembled with the *F*_*n*,1_ structures to form whole 3-shell trees ((shell-0 + shell-1) + (shell-2 + shell-3)).

Figure 1b shows representative atomic force microscopic (AFM) images of the synthesised FDFs in our study. For a perfect Cayley tree-like FDF *F*_*n*,i_, the number of nodes in the *k*th shell (*k* > 1) is given by *N*_*k*_ = *n*(*n*−1)^*k*−1^, thus the total node number of the FDF is given by Eq. (1)

1 $${\mathrm{N}}_{{\mathrm{total}}} = 1 + \mathop {\sum}\nolimits_{k = 1}^{k = i} {n(n - 1)^{k - 1}}$$

We found that the resulting structures under AFM (Fig. 1b and Supplementary Fig. 2) contained expected numbers of TDNs. *F*_2,1_, *F*_2,2_ and *F*_2,3_ contained 3, 5 or 7 TDNs; *F*_3,1_, *F*_3,2_ and *F*_3,3_ contained 4, 10 and 22 TDNs; *F*_4,1_, *F*_4,2_ and *F*_4,3_ contained 5, 17 and 53 TDNs, respectively. These results confirm the formation of FDFs with precise node number defined by the coordination number and iteration number of the TDNs. Given that the molecular weight (MW) of a TDN is ~90 kilodalton, the FDF with 53 TDNs (*F*_4,3_) possesses a MW of ~5 megadalton (Fig. 1c). It’s worth noting that this supersized structure is composed of only 16 distinct DNA strands, much less compared to a DNA origami structure of similar size (typically requires hundreds of DNA strands). The gel electrophoresis images and chromatograms (Supplementary Fig. 3) present discrete bands or peaks corresponding to FDFs with different shell numbers, which allows unambiguous discrimination of them via common gel electrophoresis or high-performance liquid chromatography (HPLC). Moreover, we found that for multi-shell FDFs, e.g., *F*_3,3_, the fractal assembly strategy led to a higher yield (84%) of end product in the final step, compared to the shell-by-shell strategy (45%, see Supplementary Fig. 4). The self-similarity of FDFs facilitates the fabrication of supersized DNA nanomaterials with minimal use of DNA strands.

### Structural properties of FDFs

**Fig. 2:**
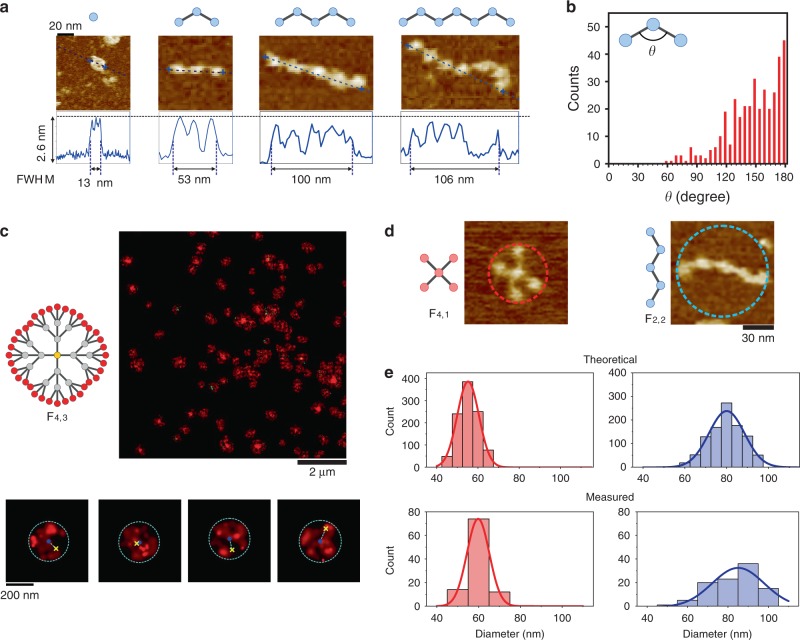
FDFs with topologically engineered structural compactness. **a** Schematic structures of *F*_2,i_ (*i* = 0-3) with representative atomic force microscopic (AFM) images and line profiles across both terminal tetrahedral DNA nanostructures (TDNs) (marked with blue cross) for *F*_2,i_. The full width of half maximum height (FWHM) are measured from the line profiles. **b** Histogram statistics of the inter-node angles in *F*_2,i_ (*N* = 360). **c** Schematic structures of *F*_4,3_ and their stochastic optical reconstruction microscopic (STORM) images (large view and individual speckles magnified from the upper image). Red speckle, Gaussian speckle (surrounded by blue dashed circle) resulted from Alexa647 labelled on the outer-shell TDNs. Yellow cross, fluorescence location of the root TDN (Cy3). Blue dot, geometric centre of the circle. **d** Schematic and representative AFM images of *F*_4,1_ and *F*_2,2_. **e** Diameter distribution of *F*_4,1_ and *F*_2,2_ from theoretical simulations (*N* = 1000) and AFM measurements (*N* = 100). Curves are Gaussian fittings of the histograms.

We next investigated the structural rigidity of FDF structures. We observed that among different FDFs, the TDNs under AFM exhibit a uniform size with apparent lateral length of ~12 nm and height of ~2.6 nm, which are consistent with that of monomer TDNs. Hence, TDNs remain intact and structurally rigid in FDFs. However, we found that the *F*_2,3_ structures generallly adopted curved conformations, resulting in shorter lateral lengths than the corresponding contour lengths (Fig. 2a). Histogram analysis shows the broad inter-node angle distribution of *F*_2,n_ ranging from 50° to 180° (Fig. 2b), suggesting these chain-like *F*_2,3_ structures have a high degree of flexibility, albeit with rigid nodes. This feature allows the construction of rigid-yet-flexible DNA materials.

We also characterised the flexibility of a dendrimer-like FDF (*F*_4,3_) in solution using stochastic optical reconstruction microscopy (STORM, detailed in Supplementary Methods). The outer-shell (shell-3) nodes of the *F*_4,3_ were labelled with Alexa647 (A647) while the root node was labelled with Cy3. As shown in Fig. 2c, we observed flower-like Guassian speckles of A647 (reconstructed from 30,000 frames of imaging), suggesting the quasi-spherical morphology of the *F*_4,3_ structures in solution. We also found that the fluorescence locations of the root nodes in some extent deviate (79 ± 36 nm, Supplementary Fig. 5) from the geometric centres of the A647 speckles, suggesting the flexible feature of the *F*_4,3_ structure.

We then asked whether the structural rigidity of FDF structures can be tuned via topological engineering (i.e. engineering their internode connection arrangements). To answer this question, we compared the structure of a chain-like FDF (*F*_2,2_) and a 4-branched FDF (*F*_4,1_). They have identical node number (five nodes) but different topological arrangements (i.e., two-way connections vs. four-way connections, shown in Fig. 2d). In *F*_2,2_, the two terminal nodes are separated by four flexible linkages and three nodes; while in *F*_4,1_, any two terminal nodes are across two linkages and one node. Thus, they should have different mechanical properties. Indeed, we found that under AFM (Fig. 2d, e and Supplementary Figs. 6 and 7), the *F*_2,2_ structures present larger size and larger size deviation (83 ± 11 nm in diameter) compared to the *F*_4,1_ structures (59 ± 5 nm), in agreement with the molecular dynamic simulations (*F*_2,2_, 80 ± 8 nm, *F*_4,1_, 59 ± 7 nm, see Methods). These results indicate that the *F*_4,1_ structure is relatively compact and rigid while the *F*_2,2_ structure is loose and flexible. This difference can be attributed to their different topologies for organising TDNs.

### FDFs encode quantised fluorescence states

**Fig. 3:**
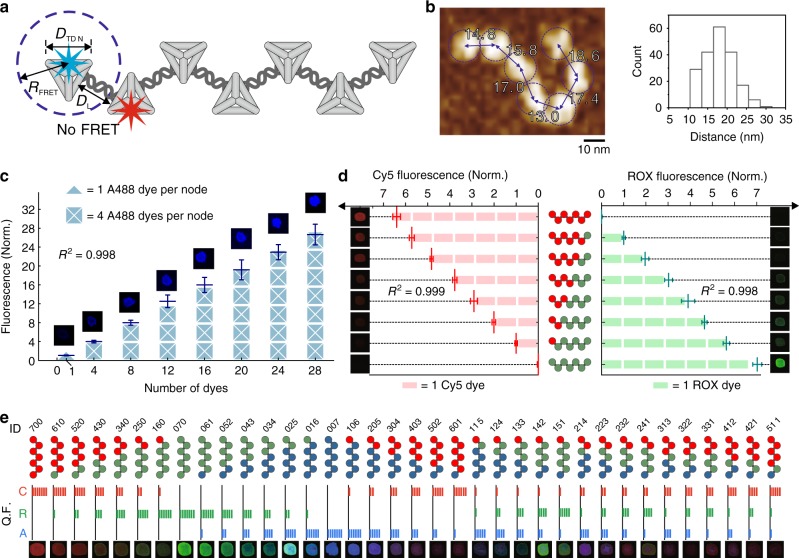
FDFs encode quantised fluorescence states. **a** Schematic illustration of the FDF structure with spatial control of fluorophores. **b** Representative AFM image of a two-way FDF (*F*_*2,3*_) structure, and statistical distribution of the internode distance of the *F*_2,n_ structures (*N* = 200). TDNs are marked with dashed circles. Measured distances between nodes (from centre to centre) are marked with numbers. **c** Correlation between fluorescence intensity and the copy number of A488 on the two-way FDF. Embedded images are fluorescence images of droplets containing corresponding FDFs in pseudocolour. **d** Correlation between fluorescence intensity and the copy number of ROX and Cy5 anchored on the *F*_2,3_ structures. Embedded images are fluorescence images of droplets containing corresponding FDFs in pseudocolour. Error bars indicate standard deviations from three independent tests. **e** 36 FDF barcodes encoded with different fluorophore combinations, which can be decoded by fluorescence spectroscopy/microscopy. The three-digit IDs refer to the numbers of Cy5, ROX and A488 in a single FDF structure (e.g., “124” refers to the presence of 1 Cy5, 2 ROX and 4 A488).

Taking advantage of the rigid-yet-flexible FDFs, we further test whether fluorophores can be precisely organised on these scaffolds to generate quantised fluorescence with minimised crosstalk (Fig. 3a). The location of each TDN node in the FDFs was measured. The lateral distance between two adjacent TDNs is 18.5 ± 3.8 nm under AFM (Fig. 3b), which is consistent with the result obtained by super-resolution stochastic optical reconstruction microscopy (STORM) at the single-molecule level (Supplementary Fig. 8), showing that the distance between two fluorophores individually anchored on two terminal TDNs of an *F*_2,1_ was ~35 nm, suggesting that two connected TDNs should be at least ~17 nm apart. Meanwhile, any two non-connected yet proximal TDNs in a folded FDF structure or in different structures were also apart for over 15 nm (Supplementary Fig. 9) Moreover, we synthesised an *F*_2,3_ structure carrying three Cy3 and four Cy5 (one fluorophore per node) for a Förster resonance energy transfer (FRET) analysis. Although the Cy3-Cy5 pair typically exhibit energy transfer when brought close to one another, we found that its fluorescence spectra were similar to that of a simple mixture of [TDN-Cy5]_3_ and [TDN-Cy3]_4_ (Supplementary Fig. 10), indicating that the distance between Cy3–Cy5 pair is beyond the effective FRET distance (>10 nm). In contrast, if the Cy3 and Cy5 were placed on the same TDN (with a distance of ~4 nm, Supplementary Fig. 10), a significant Cy3–Cy5 FRET effect was observed (quenching of Cy3 along with excitation of Cy5), in agreement with previous studies^35,47^.

Next, we prescribed four anchoring sites on each TDN node in the *F*_2,3_ structure, which can accommodate up to 28 fluorophores. By anchoring 4–28 molecules of Alexa488 (A488, max emission wavelength = 517 nm) on *F*_2,3_, we synthesised a series of FDFs with amplified fluorescence. Remarkably, the measured fluorescence intensity of the tree increases nearly linearly with the copy number of A488 (*R*^2^ > 0.99) (Fig. 3c). In particular, *F*_2,3_ with a copy number of 28 exibits ~24-fold increase in brightness as compared to a single A448 anchored on a monomeric TDN (Fig. 3c and Supplementary Fig. 11), suggesting that the ~4-nm interspace in a TDN is adequate to avoid self-quenching of A488 fluorophores, making FDF a quantitative amplifier for high-brightness fluorescence imaging. We further evaluated the fluorescence crosstalk among differently-coloured fluorophores in an FDF structure. Three types of fluorophores with distinct emission wavelengths (Cy5, 668 nm; ROX, 606 nm; A488, 517 nm, see Supplementary Fig. 12) were anchored onto FDFs with up to seven nodes, one fluorophore per node. Regardless of the presence of other types of fluorophores, the fluorescence intensity in each colour channel is proportional to the number of the corresponding fluorophores (*R*^2^ > 0.99, Fig. 3d and Supplementary Fig. 13). Taken together, FDFs enable tailorable spatial separation of fluorophores. For probes carrying heterogenous fluorophores with a larger Förster radius (i.e. the inter-fluorophore distance with 50% efficiency of FRET)^48^, the sparse fluorophore arrangement (one fluorophore per node) maintains an inter-fluorophore distance of >10 nm, thus minimising FRET quenching to ensure the linearity of fluorescence. For probes carrying a single species of fluorophores with a smaller Förster radius (typically < 4 nm), the dense arrangement (up to four fluorophores per node) can be used to realise high brightness.

According to Cayley’s formula in graph theory^49^, the FDFs have high data capacity resulting from the diversity of node combinations. To demonstrate this capacity, a set of 36 FDFs (*F*_2,3_) with all possible combination (C_3_^1^+C_3_^2^‧C_6_^1^+C_3_^1^‧C_5_^1^) of Cy5/ROX/A488 fluorophores were constructed (Fig. 3e). Each FDF is given a three-digit colour ID corresponding to the copy numbers of Cy5, ROX and A488, respectively (see characteristic fluorescence spectra in Supplementary Fig. 14). The confocal fluorescence images (Fig. 3e and Supplementary Fig. 15) show a palette comprised of 36 pseudocolours. Likewise, we constructed a series of *F*_3,1_ structures encoding 15 colours, and *F*_4,1_ structures encoding 21 colours, respectively (Supplementary Figs. 14 and 15). These colours are beyond the conventional multiplexing limit of fluorescence imaging (typically 4–5 colours).

**Fig. 4:**
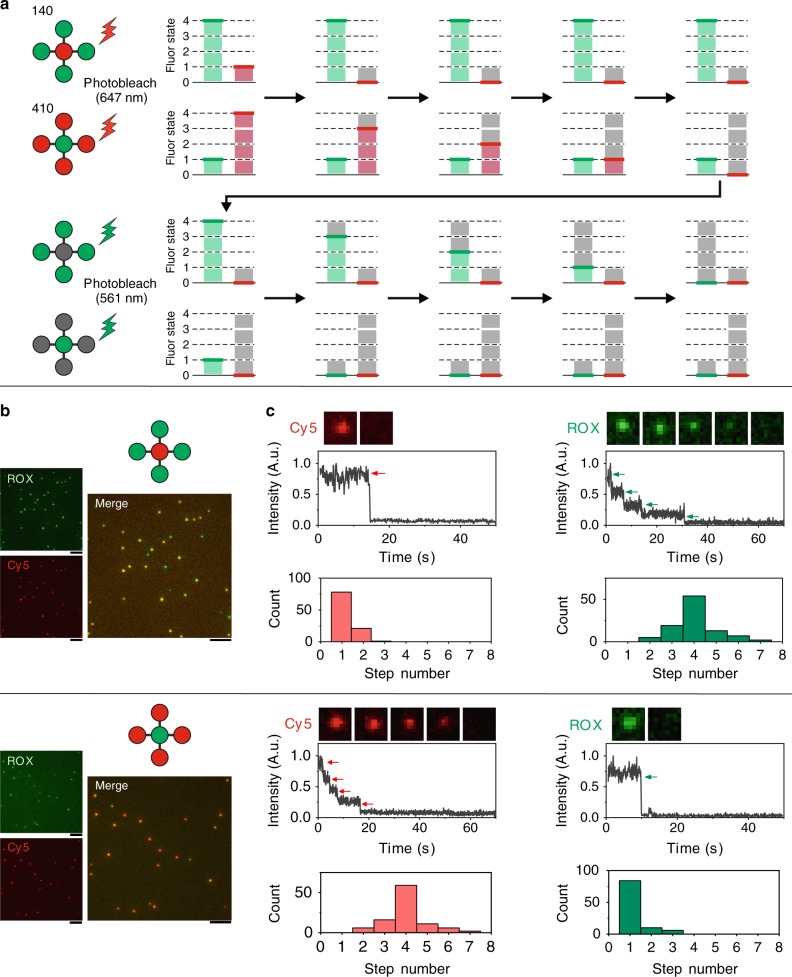
Decoding FDF barcodes via quantised photobleaching. **a** Schematic illustration of the quantised photobleaching of different FDF barcodes (ID: 140 and 410). **b** Fluorescent microscopic images of the FDF barcodes. Scale bar, 5 µm. **c** Single-molecule fluorescence quenching snapshots, kinetics of the FDF barcodes, and distributions of quenching step counts (*N* = 100 for each barcode).

We next applied the FDF-encoded barcodes as probes to single-molecule recognition and imaging. The key to this goal is to decode the fluorescence signal of a single barcode molecule, i.e. determine the number and type of fluorophores on a barcode of a specific colour. Given the fact that the fluorescence states of a barcode are quantised, the fluorescence under high-power excitation should present a stepwise photobleaching trace, as illustrated in Fig. 4a. A barcode can be decoded by the number of steps in the photobleaching trace, which correspond to the copy number of fluorophores^50–52^.

To validate this decoding strategy, we studied the photobleaching traces of two FDF barcodes (barcode ID: 140 and 410, both with Cy5 and ROX but in different ratios, as shown in Fig. 4b) with total internal reflection fluorescence microscopy (TIRF). In the initial state, spots with different fluorescence ratios from both Cy5 channel and ROX channel can be monitored (Fig. 4b). These fluorescent spots confirmed the location of the barcode molecules. Then, selected spots were illuminated by the 647-nm laser and the fluorescence kinetics was monitored within 70 s in the Cy5 channel (Fig. 4c, *N* = 100 for each type of barcodes). For barcode 140, descending fluorescence traces (78%) with one step were observed corresponding to one Cy5 fluorophore. While for barcode 410, we observed in majority (59%) four-step photobleaching traces, indicating that there were four Cy5 fluorophores present. Next, we used the 561-nm laser for excitation and photobleaching of ROX (Fig. 4c). Barcode 140 (54%) presented four-step fluorescence quenching traces while barcode 410 (84%) presented one-step traces, in accordance with their prescribed ROX numbers. These results confirmed that the quantised FDF barcodes can be decoded at the single-molecule level via photobleaching and single-particle fluorescence tracking. Although errors may occur in fluorophore counting, we estimated that over 80% barcodes can be correctly decoded (Supplementary Fig. 16), and the decoding errors might be further suppressed by choosing subsets of barcodes with high fluorescence intensity ratios between different colours.

**Fig. 5:**
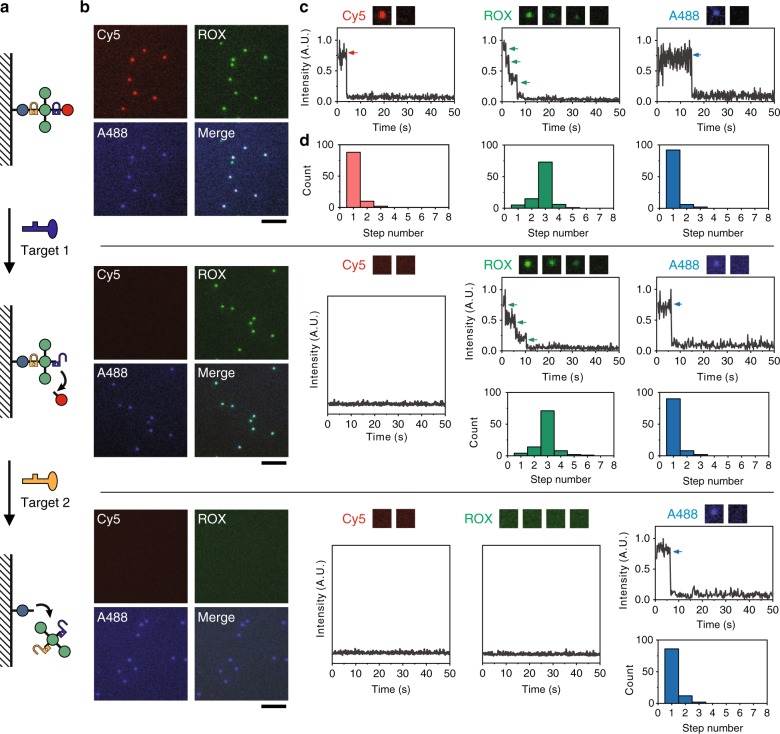
Single-molecule recognition using FDF probes. **a** Schematic illustration of the molecule recognition strategy. **b** Fluorescent microscopic images of the FDF probes. Scale bar, 5 µm. **c** Single-molecule fluorescence quenching kinetics of the FDF probes in respond to different targets. **d** Distributions of quenching step counts (*N* = 100 each).

For a single-molecule recognition experiment, an FDF probe (barcode ID: 131) was constructed to respond to specific DNA targets (Supplementary Table 1). As illustrated in Fig. 5a, two probe sequences (complementary to two DNA targets) were separately placed in two linker regions (one between ROX-TDN and Cy5-TDNs, the other between A488-TDN and ROX-TDN) of the FDF structure. The presence of these specific targets could thus trigger toehold-mediated strand-displacement reactions and break these linkers, resulting in “quantised” release of certain TDN nodes (Supplementary Fig. 17). We tracked the single-molecule fluorescence kinetics in response to the target inputs (Fig. 5b–d). Without any input, the counts of fluorescence photobleaching steps in all three channels matched the corresponding fluorophore numbers of the probe (88%, 73% and 92% of the FDFs presented one-, three- and one-step quenching traces for Cy5, ROX and A488, respectively. *N* = 100 each). After the input of target 1, we observed that the fluorescence of Cy5 disappeared, while the stepped photobleaching kinetics of A488 and ROX remained, indicating that the target 1 solely induced the specific release of Cy5-nodes. After the input of target 2, we observed the disappearance of both Cy5 and ROX fluorescence, leaving only the A488 showing a one-step photobleaching kinetics, indicating that the presence of target 2 triggered the release of ROX-nodes. In contrast, when we challenged the probe with target 2 first, the fluorescence signals of Cy5 and ROX were lost (showing no quenching steps), leaving mostly a one-step quenching trace of A488, indicating the breaking of the linkage between A488-TDN and ROX-TDN. As expected, this result remained unchanged with the subsequent input of target 1 (Supplementary Figs. 18 and 19). Collectively, using the quantised FDFs, we demonstrate a multi-state molecule recognition strategy that can translate target-induced probe cleavage into readable quantised fluorescence state changes at the single-molecule level in a sequence-sensitive manner. This strategy provides a means to develop molecular probes that can identify targets or multiple target states.

### FDF barcodes for cell imaging and discrimination

**Fig. 6:**
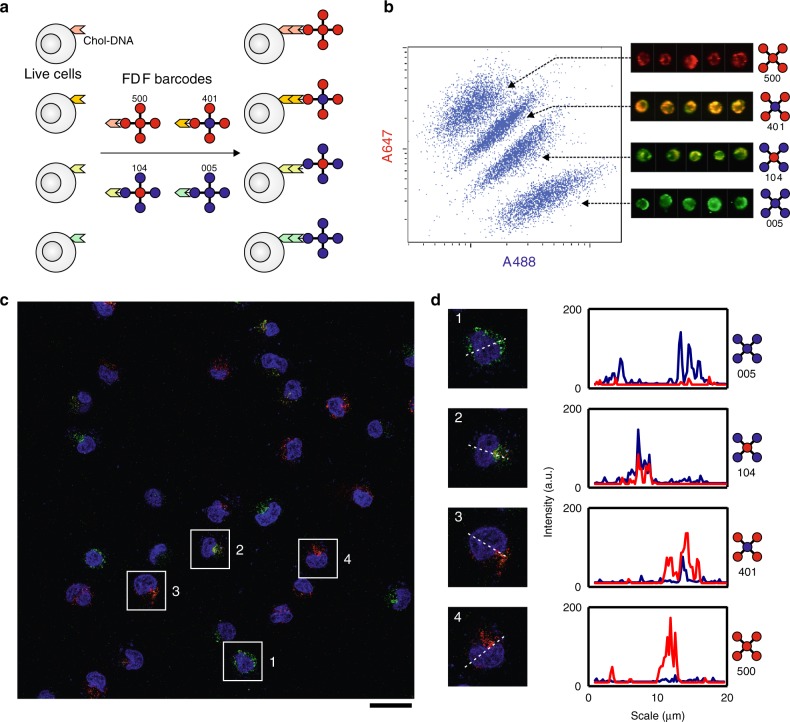
FDF barcodes for cell imaging and discrimination. **a** Schematic illustration of FDF barcodes for discrimination of cell populations. **b** Cytometric scatter plot and representative snapshots of FDF-tagged cells during cell sorting. **c** Wide-view fluorescence image of FDF-tagged cells and **d** representative fluorescence intensity profiles (along the dashed lines in the cell images). Scale bar, 25 μm.

Having established the FDF barcodes, we further sought to implement multiplexed cell imaging and discrimination. We found that the *F*_4,1_ structure could maintain structural stability within 12 h in serum culture and showed minimal cytotoxicity (Supplementary Figs. 20 and 21), which supports the use of FDF barcodes in live-cell imaging. We built four *F*_4,1_-based FDF barcodes (colour IDs: 500, 401, 104 and 005) carrying overhangs of distinct sequences (Fig. 6a). Four groups of HeLa cells were separately tagged with cholesterol-modified ssDNAs (chol-DNAs, which can be embedded in cell membranes and allow DNA hybridisation) orthogonally complementary to the overhangs of the FDF barcodes. Then the cells were mixed up and incubated with the mixture of the four FDF barcodes. The flow cytometric result (Fig. 6b) shows that the cell mixture could be sorted into four separated populations with different A647/A488 fluorescence intensity ratios, which characteristically correspond to the four FDF barcodes, and are in agreement with the results from separately tagged cells (Supplementary Fig. 22). In the wide-view fluorescence image (Fig. 6c), the cells can also be classified into four groups by their fluorescence intensity profiles (Fig. 6d), matching the four FDF barcodes. Taken together, these results suggest that the multiplexity of FDF barcodes allows discrimination of multiple cell populations in a cell mixture.

## Discussion

In this study, we report a topological engineering approach to construct multiplexed FDF amplifiers. The Cayley tree-like fractal assembly features simplicity, modularity and high capacity for fluorescence encoding and decoding, which compares favourably with previously reported DNA assembly methods that either require high number of DNA strands or lack well-defined structures^23,44,53–56^. Luo’s group have developed a series of dendrimer-like DNA structures based on Y-DNA, and demonstrated their use in fluorescence encoding^34,54,57^. But their structural compactness, topological engineerability and cargo/information capacity are still limited. Here, we employed tetrahedral DNA nanostructures as rigid nodes for the Cayley tree framework, which have defined coordination number (2–4), higher rigidity and molecular weights, leading to FDF structures with fixed numbers of nodes and guest molecules. By using only a small set of oligonucleotides (as few as 16 distinct sequences), we can construct FDFs with up to 53 TDN nodes and ~5-megadalton in molecular weight.

We show that the structural flexibility of FDFs can be topologically engineered, which allows modular design of DNA nanomaterials with tuneable mechanical properties. Moreover, these nodes have large information capacity with minimal cross-talk. By individually anchoring fluorophores with different emitting wavelength on each TDN node, we synthesised colour-encoded multiplexed probes for identification of different molecules and live cells. Hence, FDFs provide a highly generic amplification toolset for multiplexed and quantitative detection of low-abundance biological targets.

## Methods

### Preparation and purification of TDN nodes and the FDFs

To synthesise TDN nodes, four component oligonucleotides (S1, S2, S3 and S4, synthesised by Invitrogen, USA, sequences were listed in Supplementary Table 1) of the TDN structure were mixed in equimolar concentration (1 µM each) in TM buffer (10 mM Tris-HCl, 5 mM MgCl_2_, pH 8.0). The mixture was heated to 95 °C for 10 min, then rapidly cooled to 4 °C in a thermal cycler (Applied Biosystems Veriti 96 well Thermal Cycler). HPLC purification and characterisation were carried out using an Agilent 1260 system. An SEC column (Phenomenex BioSec-SEC-S4000, 300 × 7.8 mm) was used to purify the TDN nodes, and the chromatograms were recorded at 260 nm. The mobile phase was 25 mM Tris-HCl, pH 7.2, 450 mM NaCl with a flow rate of 1 ml min^−1^. To fabricate FDFs, the purified TDN nodes with complementary linking strands were mixed in equimolar concentration (0.5 µM each) and incubated at 25 °C for 2 h. The products were again purified with the SEC column.

### AFM imaging

AFM images were taken with a Bruker multimode 8 atomic force microscope (Veeco Inc., USA). Freshly cleaved mica was treated with a 0.5% (v/v) APTES (Sigma-Aldrich) for 2 min, washed with Milli-Q water (18 MΩ cm), and dried with compressed air prior to nanostructures deposition. In all, 10 nM of the the sample in 30 µl TM buffer was deposited on the mica surface and let sit 5 min for adsorption. The samples were imaged in tapping mode in solution using SNL-10 tips.

### Fluorescence spectroscopic measurements

All fluorescence spectroscopic measurements were carried out using an Edinburgh FS920 fluorescence spectrometer operating in photon counting mode. The fluorescence emission spectra of Alexa488 labelled structures were collected from 495 to 590 nm with excitation at 488 nm. The fluorescence emission of ROX was collected from 595 to 640 nm with excitation at 588 nm. The fluorescence of Cy5 was collected from 655 to 720 nm by with excitation at 650 nm. All spectra were collected at 25 °C.

### Confocal microscopic imaging

For multi-colour FDF fluorescence imaging, solutions of FDFs were dropped onto the slides to form 6 × 6 Dot Matrix shapes using a SpotBot 2 microarrayer programmed by SpotApp software (ArrayIt SpotBot 3). Then, confocal microscopic imaging of these spots was carried out on a laser confocal microscope (Leica TCS SP8) at 10× magnification. For Cy5, exitation (ex) 647 nm, emission (em) 650–720 nm; For ROX, ex 561 nm and em 575–620 nm. For A488, ex 488 nm, em 495-550 nm.

### Single molecule imaging

FDF barcodes (ID:140 or 410, 20 pM) were adsorbed on clean glass coverslips respectively for 10 min. Total internal reflection fluorescence (TIRF) imaging was performed on a commercial super-resolution microscope (N-STORM, Nikon) with a 100× objective lens (NA 1.49) and an electron multiplying charge-coupled device (EMCCD) camera (Andor, iXon 3). The FDF barcodes were first excited with 647-nm solid state laser for 70 s. The second round of excitation was carried out by using 561-nm laser for 70 s. The fluorescence intensity kinetics were analysed from the fluorescence spots across the entire movie through Nikon N-STORM analysis software.

For single-molecule recognition, a clean glass coverslip was modified with the mixture of biotin-PEG and PEG as described previously^58^. Streptavidin was then adsorbed on the coverslip via biotin-avidin interaction. Next, the biotinylated FDF single-molecule probe (ID: 131, 20 pM) was immobilised on the coverslip. Then, a target strand was added into the solution (final concentration, 100 pM). After an incubation at 25 °C for 30 min, the sample was washed with 1× PBS three times. The target-triggered strand-displacement reaction was verified using gel electrophoresis (Supplementary Fig. 16). 647-nm, 561-nm and 488-nm solid state laser (Coherent) were used to excite Cy5, ROX and A488 (70 s each), successively. The fluorescence intensity kinetics of the fluorescent spots were recorded.

### Cell imaging and discrimination

For cell discrimination, four *F*_4,1_-based barcodes loaded with different ratios of A647 and A488 (colour ID: 500, 401, 104 and 005) were built. Each of them carried a distinct overhang sequence (sequences listed in Supplementary Table 1). For cell labelling, four groups of HeLa cells (~6 × 10^4^ cells each group) were separately incubated (for 0.5 h at 37 °C) with cholesterol-modified ssDNAs (chol-DNAs, sequences listed in Supplementary Table 1) orthogonally complementary to the barcodes. The cells were washed with PBS solution for three times to remove the free chol-DNAs. Next, the four groups of cells were trypsinized and mixed up in serum-free culture medium and incubated with the mixture of the FDF barcodes (chol-DNAs) for 1 h at 37 °C. These cells were washed three times with PBS and centrifugation at 1000 rpm, and resuspended in 100 μL of PBS. The cell mixture was underwent flow cytometry analysis using a Merk Millipore amins Image streamX Mark II system (A647 channel, Ex 642 nm and Em 642–745 nm. A488 channel, Ex 488 nm and Em 505–560 nm). Confocal imaging of these cells was carried out on a Leica TCS SP8 confocal laser scanning microscope. The cells nuclei were stained with Hoechst33258 for 10 min prior to imaging. For A647 channel, Ex 633 nm and Em 650–720 nm; A488 channel, Ex 488 nm and Em 495-550 nm; Hoechst channel, Ex 405 nm and Em 420–470 nm. The fluorescence profiles were extracted and analysed using ImageJ.

## Supplementary information

Supplementary Information

## Data Availability

The data that support the findings of this study are available within the paper and its Supplementary Information and are available from the corresponding authors upon reasonable request.
