# Supplementary material for: Encoding quantized fluorescence states with fractal DNA frameworks
Source: Nat Commun. 2020 May 4;11:2185. doi: 10.1038/s41467-020-16112-z (PMC7198603; doi:10.1038/s41467-020-16112-z)
Supplement: Supplementary file 1 — Supplementary Information [file 41467_2020_16112_MOESM1_ESM.pdf]

Supplementary information for

Encoding quantized fluorescence states with fractal DNA frameworks

Jiang Li[1,2†], Jiangbing Dai[2†], Shuoxing Jiang[3†], Mo Xie[2†], Tingting Zhai[4], Linjie Guo[2], Shuting Cao[2], Shu Xing[2], Zhibei Qu[4], Yan Zhao[2], Fei Wang[4], Yang Yang[2], Lei Liu[2], Xiaolei Zuo[4], Lihua Wang[1,5*], Hao Yan[3*], and Chunhai Fan[4*]

[1]Bioimaging Center, Shanghai Synchrotron Radiation Facility, Zhangjiang Laboratory, Shanghai Advanced Research Institute, Chinese Academy of Sciences, Shanghai, 201204, China
[2]Division of Physical Biology, CAS Key Laboratory of Interfacial Physics and Technology, Shanghai Institute of Applied Physics, Chinese Academy of Sciences, Shanghai 201800, China
[3]Center for Molecular Design and Biomimetics, The Biodesign Institute, School of Molecular Sciences, Arizona State University, Tempe, AZ, 85287, USA
[4]Frontiers Science Center for Transformative Molecules, School of Chemistry and Chemical Engineering, Institute of Molecular Medicine, Renji Hospital, School of Medicine, Shanghai Jiao Tong University, Shanghai 200024, China
[5]Shanghai Key Laboratory of Green Chemistry and Chemical Processes, School of Chemistry and Molecular Engineering, East China Normal University, 500 Dongchuan Road, Shanghai, 200241, China
†These authors contributed equally.
*E-mail: wanglihua@sinap.ac.cn; hao.yan@asu.edu.cn; fanchunhai@sjtu.edu.cn

Supplementary Methods

HPLC characterization and purification. HPLC purification and characterization were carried out (for TDN nodes and FDF structures with no more than 7 nodes) on an Agilent 1260 system. An SEC column (Phenomenex BioSec-SEC-S4000, 300 × 7.8 mm) was used to characterize and purify the TDNs and some of the FDFs ($F_{2,n}$, $F_{3,1}$, and $F_{4,1}$). Chromatograms were recorded at 260 nm. The mobile phase was 25 mM Tris-HCl, pH 7.2, 450 mM NaCl with a flow rate of 1 ml min^{-1}.

Gel characterization and purification. For gel characterization and purification of TDNs, the PAGE electrophoresis was carried out in an 8% polyacrylamide gel (acrylamide/bis-acrylamide ratio, 29:1) using a Bio-Rad vertical gel electrophoresis system (typically 120 V, 100 min). For FDFs, the electrophoresis was carried out in an agarose gel (BioRad, 1% w/v) using a Bio-Rad horizontal gel electrophoresis system (typically 85 V, 60 min). The loading buffer contained 50% glycerol and colour tracker (Bromphenol Blue and Xylene Cyanol FF). The electrophoresis buffer was 1× TBE (tris-borate-EDTA). After electrophoresis, the gel was stained with Gel Red (Biotium, USA) following the protocol provided by the manufacturer.

STORM imaging of FDFs. STORM Imaging (N-STORM super-resolution microscope, Nikon) was performed using inclined illumination with excitation intensity of ~200 W/cm^2 at 405nm, 561nm or 647 nm, following the protocols provided by the manufacturer. All images were reconstructed from more than 15000 frame long time-lased movies acquired with 20 ms integration time. For monocolour imaging, the images were reconstructed using spot finding and Gaussian fitting algorithms with ImageJ software. Fluorescent microspheres were used as fiducial markers for drift correction by tracking the position of each marker, and performed in the final super-resolution reconstruction. For multicolour imaging, Nikon N-STORM analysis software was used for image reconstruction.

Molecular Dynamics Simulations. The simulations of linear and network DNA tetrahedron structures were carried out using oxDNA with the sequence-dependent parametrization of hydrogen-bonding and stacking interactions[1, 2]. The simulations were carried out on NVIDIA GPUs using molecular dynamics (MD) simulation with an Andersen-like thermostat and simulation time step of 0.009 ps[3]. The temperature was set to 20 °C. We ran the MD simulations for each of the DNA nanostructures for the number of steps

time corresponding to 30 μs. Moreover, to speed up the sampling of different conformations, a diffusion coefficient that corresponds to 7.6×10^{-8} m^2/s of a 14 bp duplex in the simulation was applied, which corresponds to approximately 600 times faster diffusion than observed experimentally[4]. To obtain the average size for each nanostructure and calculate the mean deviation, we saved 1000 different conformations from the MD simulation. We then randomly picked one structure from the ensemble and aligned all of the remaining structures onto this one so that the root-mean-square distance between the centers of mass of all corresponding nucleotides is minimized.

Decoding accuracy estimation. To evaluate the reliability of barcode decoding, we established an estimation model based on computer-generated samples bearing random errors. Take the 7-node FDF barcodes as an example. We first used MatLab to generate a large number of three-dimensional vectors to represent the barcodes (36 barcode species, N=1000 each) with combinations of three fluorophore species (e.g., a vector [1, 1, 5] indicates the barcode with 1 Cy5, 1 ROX, and 5 A488 dyes). Considering our experimental observation, we assumed that each fluorophore on the barcodes has a 5% chance of being lost in fluorophore counting and a 1% chance of being overcounted. These variations with given probabilities were introduced into the computer-generated vectors, mimicking real barcode samples with errors in fluorophore counting.

Next, the vectors of these computer-generated samples were identified one by one by using MatLab. Briefly, if a sample vector matches any of the standard barcodes, it is marked as "matched" (Supplementary Fig. 16). Otherwise, it is "unmatched". Among the matched samples, there were still a few that matched to incorrect barcodes ("matched incorrectly"). Whereas, a part of unmatched samples could still be correctly identified via the Cosine Similarity analysis given by Equation (1):

$$\cos\theta = \frac{\overrightarrow{sample} \cdot \overrightarrow{reference}}{|sample| \cdot |reference|} \quad (1)$$

where "sample" refers to a sample vector that needs to be identified; "reference" indicates the vector of a standard barcode; and θ is the angle between them in their vector space, which reflects their similarity (a bigger cos θ indicates a higher similarity).

The samples were calculated with reference barcodes using this equation. The reference barcode resulting

in the maximal cos θ is regarded as the estimated answer of a given sample. If the estimated answer is correct ("estimated correctly"), it is also regarded as being correctly decoded.

Analysis of stability in cell medium. To analyze the serum stability of FDFs, these constructs were suspended in a solution comprising 10% (v/v) fetal bovine serum (FBS) in 1640 culture medium to achieve a solution with 10 nM final structure concentration at 37 °C. The resulting mixture was incubated at 37 °C and 20 µl aliquots were collected after 2, 8, 12, and 24 h for analysis by gel electrophoresis.

Cell Culture. Hela and cells were separately grown in MEM medium (Invitrogen) supplemented with 10% (v/v) FBS, 1% penicillin/streptomycin, and 2 mM L-glutamine at 37 °C in humidified air containing 5% CO_2.

Cytotoxicity evaluation. The cytotoxicity of FDFs was assessed by MTT assay. HeLa were dispersed within a 96-well plate at a concentration of 1×10^5 cells per well and incubated at 37°C in 5% CO_2. After 24 h, the FDF was introduced into the culture media at 20 nM. Cells were incubated with the FDF for another 6, 12 and 24 h. Then, the cells were washed with PBS. 100 µl of 1×MTT solution (about 1.5 mM) was added to each well. After 4 hours, the MTT-containing media was removed, and 100 µl DMSO was added to each well to dissolve the formazan crystals. The light adsorption at 570 nm was measured using a Bio Tek Synergy MX H1 reader.

Supplementary Tables

Supplementary Table 1. Sequences used in this study.

Sequences for building TDN nodes. Underlined sequences are the linker sequences between TDN nodes.	
Name	Sequence (5’ to 3’)
L1-S1	GTGCTTGGTAACATAGGTGCACAGCCAGTTGAGACGAACATTCCTAAGTCTGAAATTTATCACCCGCCATAGTAGACGTATCACCAGG
L1-S2	GTGCTTGGTAACATAGGTGCACAGCGCTACACGATTCAGACTTAGGAATGTTCGACATGCGAGGGTCCAATACCGACGATTACAGCTT
L1-S3	GTGCTTGGTAACATAGGTGCACAGCGTGATAAAACGTGTAGCAAGCTGTAATCGACGGGAAGAGCATGCCCATCCACTACTATGGCGG
L1-S4	GTGCTTGGTAACATAGGTGCACAGCCTCGCATGACTCAACTGCCTGGTGATACGAGGATGGGCATGCTCTTCCCGACGGTATTGGACC
L1’-S1	GCTGTGCACCTATGTTACCAAGCACCAGTTGAGACGAACATTCCTAAGTCTGAAATTTATCACCCGCCATAGTAGACGTATCACCAGG
L2-S2	AAAAAAAAAAAAAAAAAAAAAAAAAGCTACACGATTCAGACTTAGGAATGTTCGACATGCGAGGGTCCAATACCGACGATTACAGCTT
L2-S3	AAAAAAAAAAAAAAAAAAAAAAAAAGTGATAAAACGTGTAGCAAGCTGTAATCGACGGGAAGAGCATGCCCATCCACTACTATGGCGG
L2-S4	AAAAAAAAAAAAAAAAAAAAAAAAACTCGCATGACTCAACTGCCTGGTGATACGAGGATGGGCATGCTCTTCCCGACGGTATTGGACC
L2’-S1	TTTTTTTTTTTTTTTTTTTTTTTTTCAGTTGAGACGAACATTCCTAAGTCTGAAATTTATCACCCGCCATAGTAGACGTATCACCAGG
L3-S2	AAAGCCGGTAAGGTGAGGAATCCGAGCTACACGATTCAGACTTAGGAATGTTCGACATGCGAGGGTCCAATACCGACGATTACAGCTT
L3-S3	AAAGCCGGTAAGGTGAGGAATCCGAGTGATAAAACGTGTAGCAAGCTGTAATCGACGGGAAGAGCATGCCCATCCACTACTATGGCGG
L3-S4	AAAGCCGGTAAGGTGAGGAATCCGACTCGCATGACTCAACTGCCTGGTGATACGAGGATG

	GGCATGCTCTTCCCGACGGTATTGGACC
L3'-S1	TCGGATTCCTCACCTTACCGGCTTTCAGTTGAGACGAACATTCCTAAGTCTGAAATTTATCACCCGCCATAGTAGACGTATCACCAGG
S2	GCTACACGATTCAGACTTAGGAATGTTCGACATGCGAGGGTCCAATACCGACGATTACAGCTT
S3	GTGATAAAACGTGTAGCAAGCTGTAATCGACGGGAAGAGCATGCCCATCCACTACTATGGCGG
S4	CTCGCATGACTCAACTGCCTGGTGATACGAGGATGGGCATGCTCTTCCCGACGGTATTGGACC
Sequences for Single-molecule recognition. T1 and T1' are for Target1-triggered disconnection; T2 and T2' are for Target2.	
T1-S4	ACCTGAGTGAGTATGCGGAGTACGACTCGCATGACTCAACTGCCTGGTGATACGAGGATGGGCATGCTCTTCCCGACGGTATTGGACC
T1'-S1	CTCCGCATACTCACTCAGGTCAGTTGAGACGAACATTCCTAAGTCTGAAATTTATCACCCGCCATAGTAGACGTATCACCAGG
T2-S3	CATCTCTGCTCTAGTGAGTCTCGAAGTGATAAAACGTGTAGCAAGCTGTAATCGACGGGAAGAGCATGCCCATCCACTACTATGGCGG
T2'-S1	GACTCACTAGAGCAGAGATGCAGTTGAGACGAACATTCCTAAGTCTGAAATTTATCACCCGCCATAGTAGACGTATCACCAGG
Target1	TCGTACTCCGCATACTCACTCAGGT
Target2	TTCGAGACTCACTAGAGCAGAGATG
Sequences for discrimination of cell populations tagged with chol-DNAs. Overhangs on FDFs (underlined) are appended on the 5' end of S4 (O_n-S4)	
Chol-DNA1	GCTGTGCACCTATGTTACCAAGCAC-chol
Chol-DNA2	GAATCCTGAAAGACCACTGTTTTGG-chol
Chol-DNA3	GCGATGCTGTTATCGATGGAGACCG-chol
Chol-DNA4	TTACTGGTGACGTGAGCGTCCTTTT-chol
O1-S4	GTGCTTGGTAACATAGGTGCACAGCCTCGCATGACTCAACTGCCTGGTGATACGAGGATG

	GGCATGCTCTTCCCGACGGTATTGGACC
O2-S4	<u>CCAAAACAGTGGTCTTTCAGGATTCC</u>TCGCATGACTCAACTGCCTGGTGATACGAGGATG<br>GGCATGCTCTTCCCGACGGTATTGGACC
O3-S4	<u>CGGTCTCCATCGATAACAGCATCGC</u>CTCGCATGACTCAACTGCCTGGTGATACGAGGATG<br>GGCATGCTCTTCCCGACGGTATTGGACC
O4-S4	<u>AAAAGGACGCTCACGTCACCAGTAA</u>CTCGCATGACTCAACTGCCTGGTGATACGAGGATG<br>GGCATGCTCTTCCCGACGGTATTGGACC

Supplementary Table 2. Sequence combinations for building TDN nodes in different FDF structures.

	Shell-0 (Root node)	Shell-1	Shell-2	Shell-3 (leaf nodes)
$F_{2,i}$	L1-S1,	L1'-S1,	L2'-S1,	L3'-S1,
	L1-S2,	L2-S2,	L3-S2,	S2,
	S3,	S3,	S3,	S3,
	S4	S4	S4	S4
$F_{3,i}$	L1-S1,	L1'-S1,	L2'-S1,	L3'-S1,
	L1-S2,	L2-S2,	L3-S2,	S2,
	L1-S3,	L2-S3,	L3-S3,	S3,
	S4	S4	S4	S4
$F_{4,i}$	L1-S1,	L1'-S1,	L2'-S1,	L3'-S1,
	L1-S2,	L2-S2,	L3-S2,	S2,
	L1-S3,	L2-S3,	L3-S3,	S3,
	L1-S4	L2-S4	L3-S4	S4

Supplementary Figures

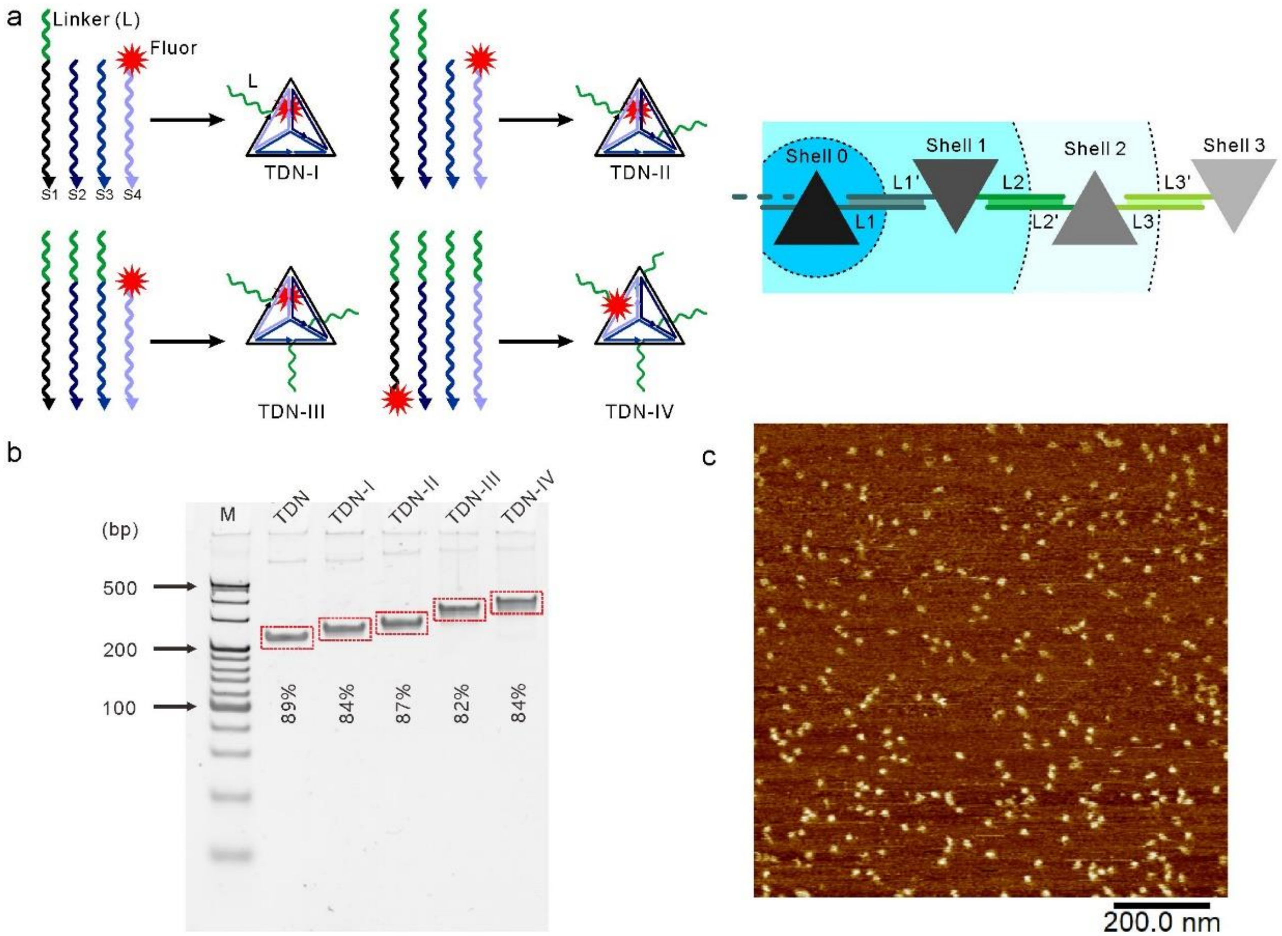

Supplementary Figure 1. Assembly of DNA tetrahedron nodes (TDNs). (a) Assembly of the TDNs with different numbers of linkers (L) appending to the 5' ends of component strands. For TDNs I-III carrying one fluorophore each, the fluorophore is labeled on the 5' end of the component strand S4 (Supplementary Table 1). For TDN-IV, the fluorophore is labeled on the 3' end of S1. (b) Native PAGE (8%) characterization of the assembly of TDNs (bands marked with red dash boxes. Numbers below are the yields of TDNs quantified from the band intensity). M: 20 bp DNA ladder. (c) AFM image of TDN-II.

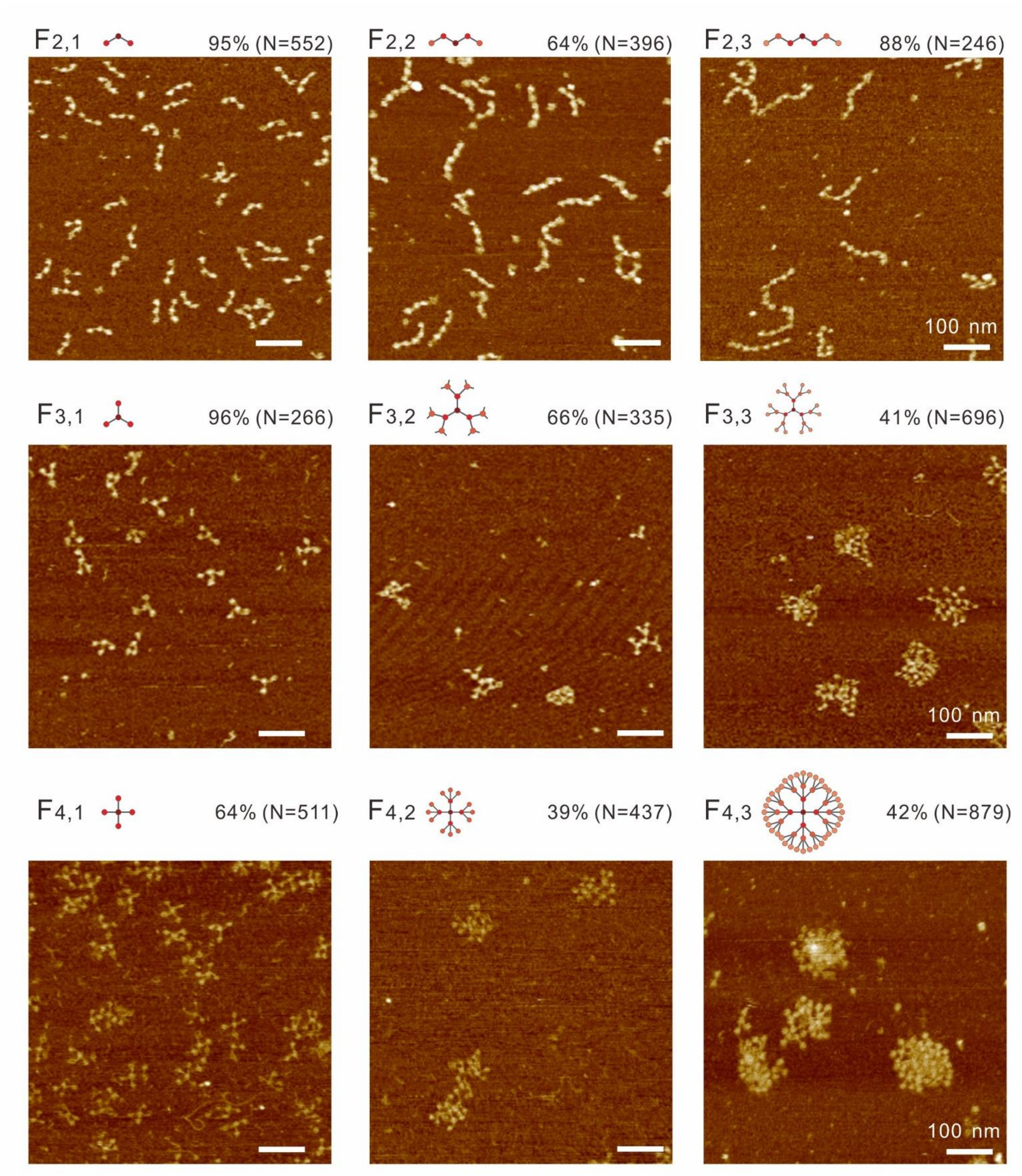

Supplementary Figure 2. Wide view atomic force microscopic (AFM) images of FDF structures. Percentage numbers, the proportion of TDNs (N=total count) that form the FDF structures with expected node numbers. Scale bar, 100 nm.

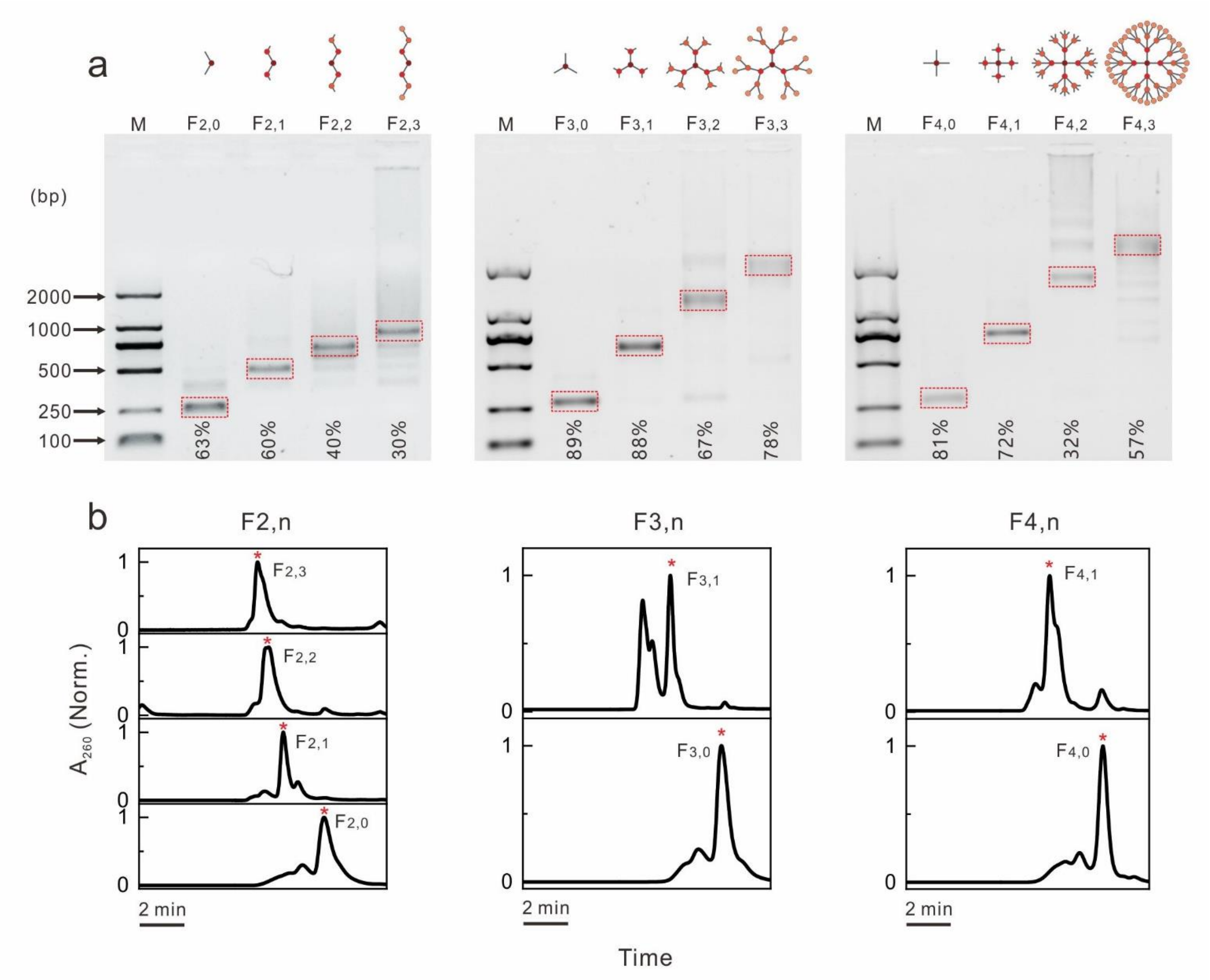

Supplementary Figure 3. Characterization of fractal DNA frameworks (FDFs). (a) Native agarose gel (1%) electrophoresis images of the FDF structures. M, DNA marker DL2000. Percentages below are yields of expected structures obtained from band intensity quantification. (b) Chromatograms (absorption at 260 nm) of some FDF structures

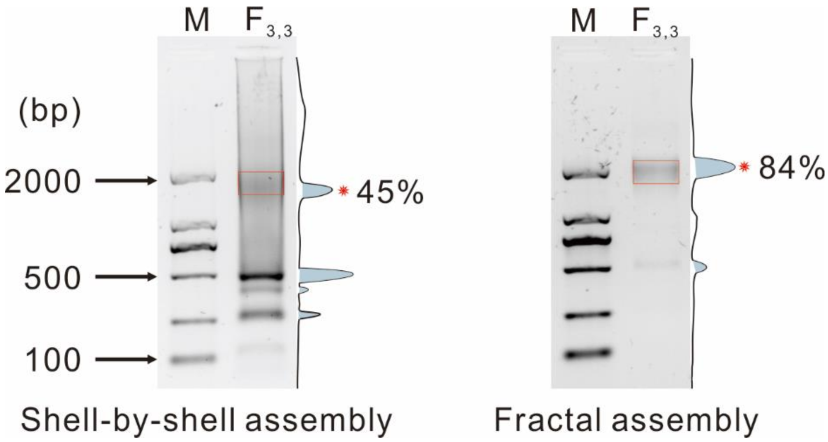

Supplementary Figure 4. Native gel electrophoresis images of the $F_{3,3}$ structures. M, DNA marker DL2000. The yield of $F_{3,3}$ structure was ~84% by using the fractal assembly strategy and was ~45% by using the shell-by-shell strategy.

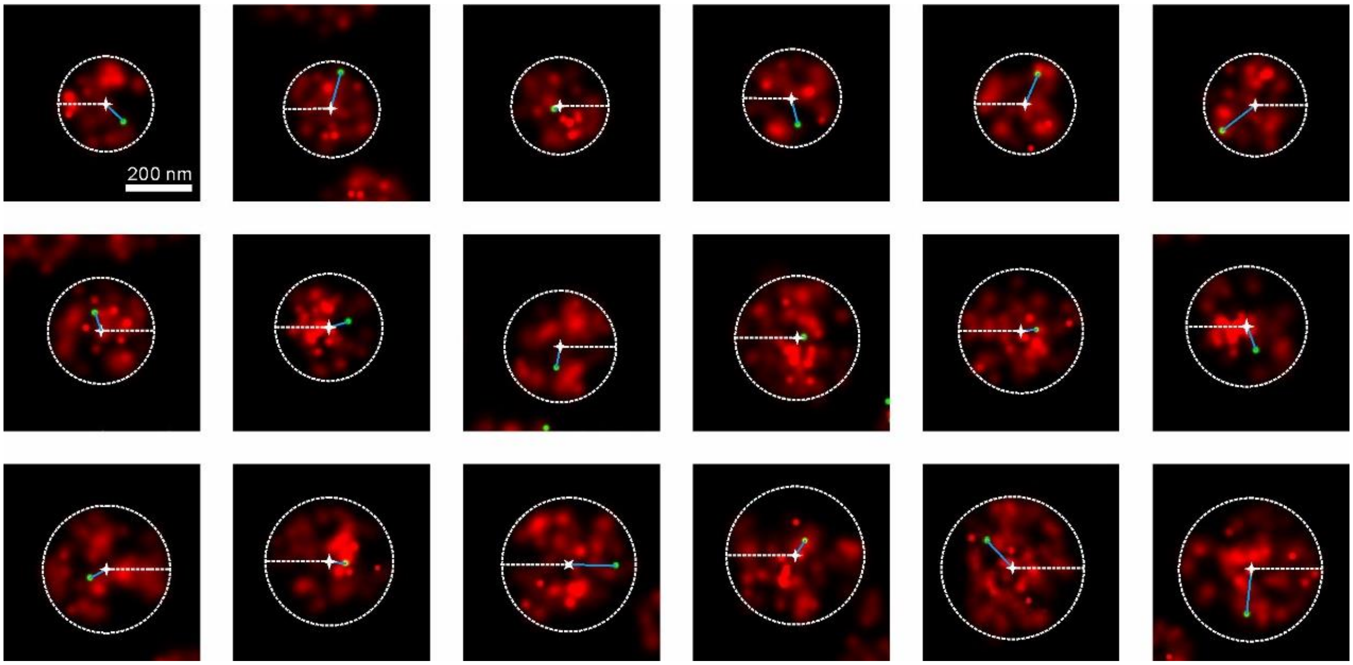

Supplementary Figure 5. Stochastic optical reconstruction microscopic (STORM) images of $F_{4,3}$ structures with A647-labelled outer-shell TDNs. Red-coloured spots, Gaussian speckles of labeled TDNs, outlined by white dashed circles with the geometric centres marked with white crosses and Cy3-labelled root TDNs marked with green dots.

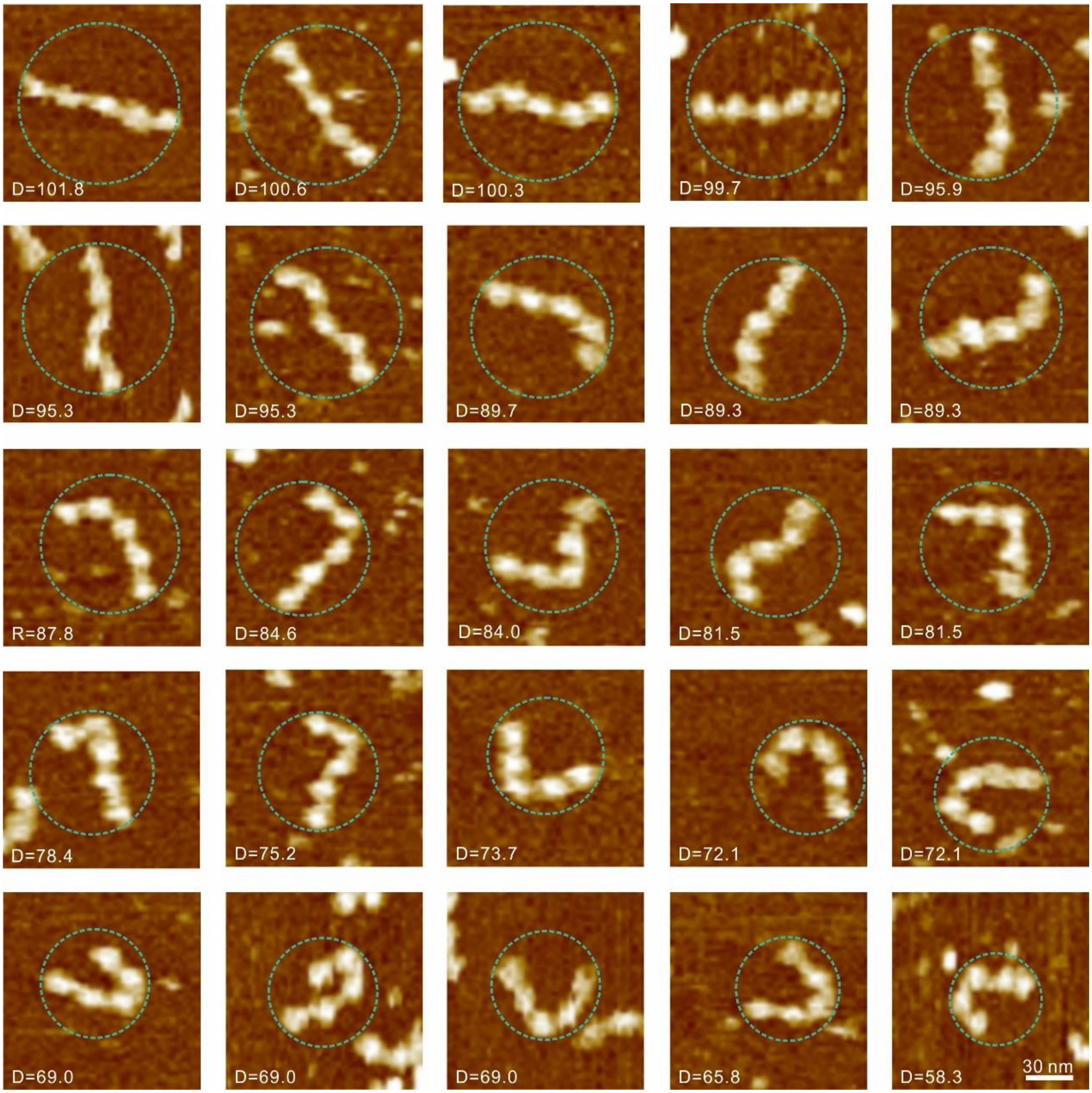

Supplementary Figure 6. Representative AFM images of the $F_{2,2}$ structures. Green dashed circles, outlines of the structures with diameters marked at the left lower. Scale bar, 30 nm.

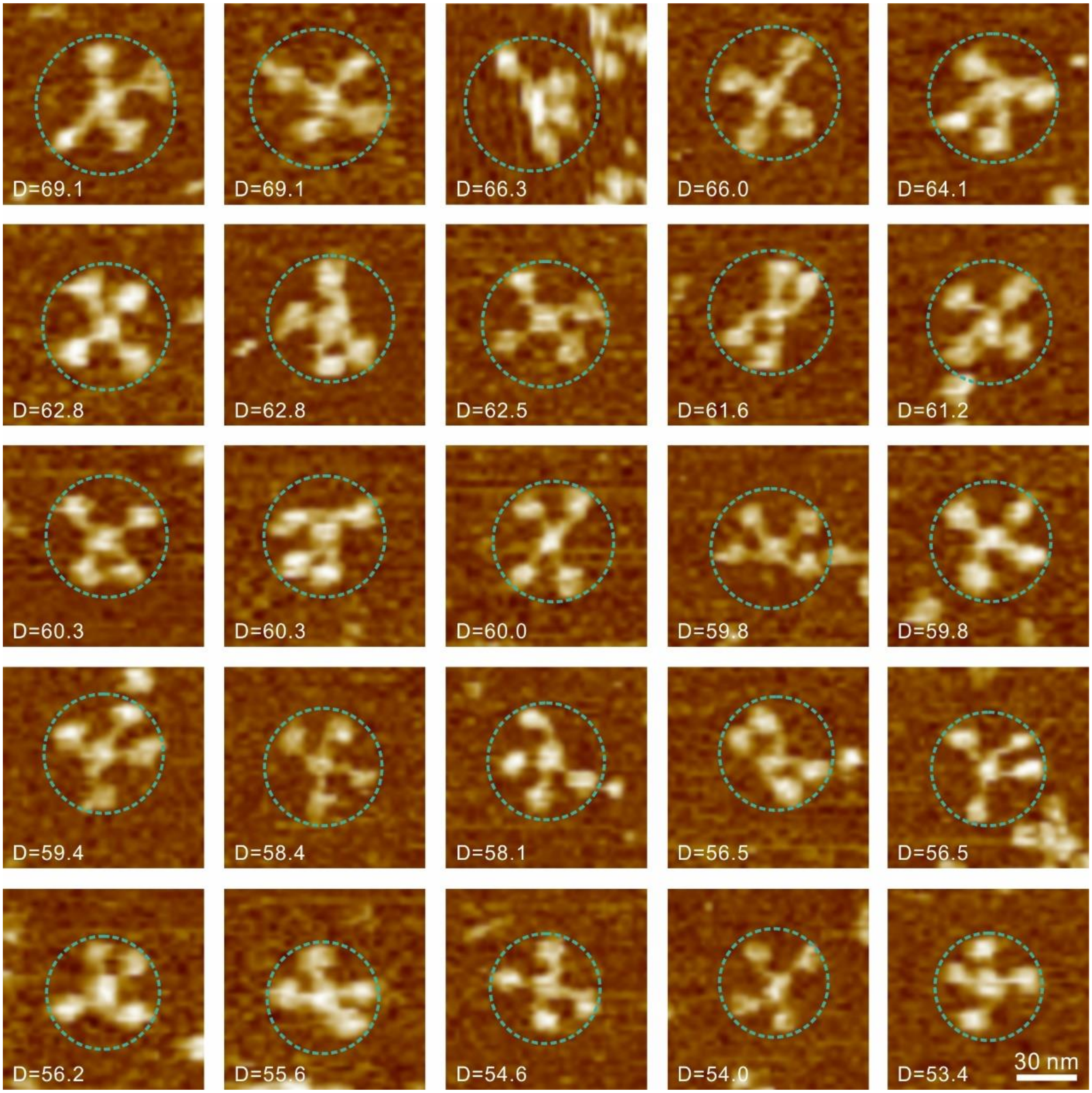

Supplementary Figure 7. Representative AFM images of the $F_{4,1}$ structures. Green dashed circles, outlines of the structures with diameters marked at the left lower. Scale bar, 30 nm.

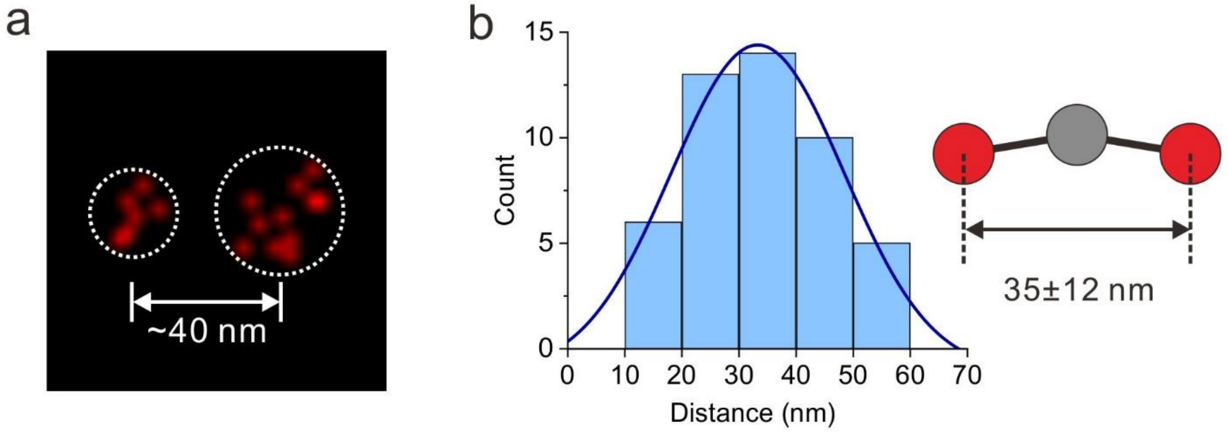

Supplementary Figure 8. Measurement of the lateral length of a F2,1 structure. (a) Rrepresentative STORM image of $F_{2,1}$. Two terminal TDNs were labeled with Alexa647 (Em, 674-786 nm). (b) Distribution of the distance between two terminal TDNs localized by STORM (N=48). Blue curve, Gaussian fitting of the distribution. Right, schematic of the $F_{2,1}$ structure and the mean distance between two terminal TDNs.

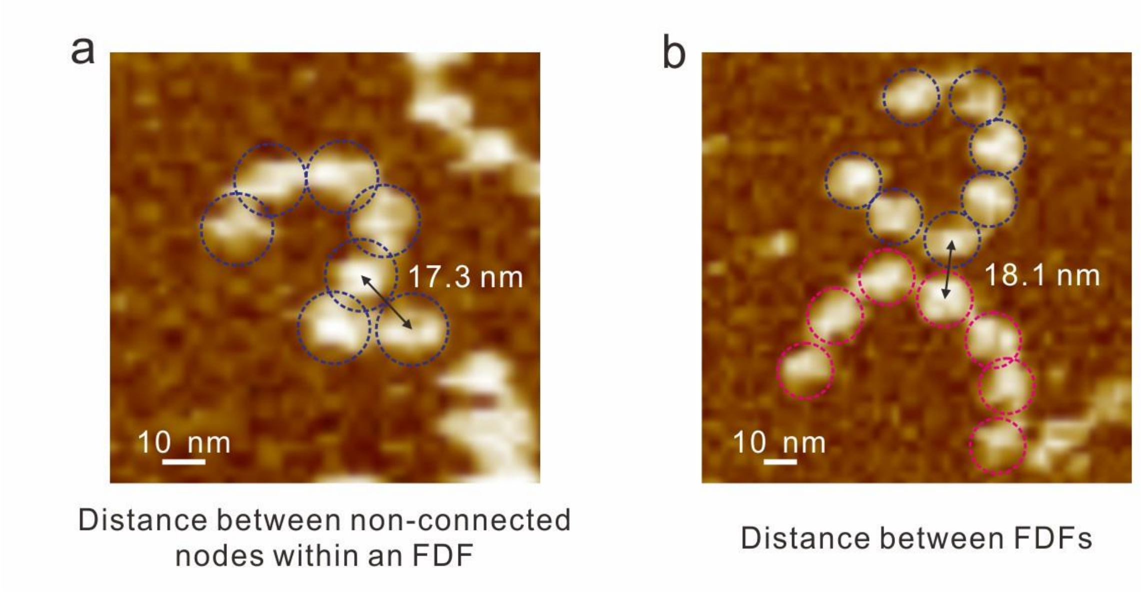

Supplementary Figure 9. AFM measurements of the internode distance. (a) Distance between non-connected nodes within an FDF. (b) Distance between different FDF structures.

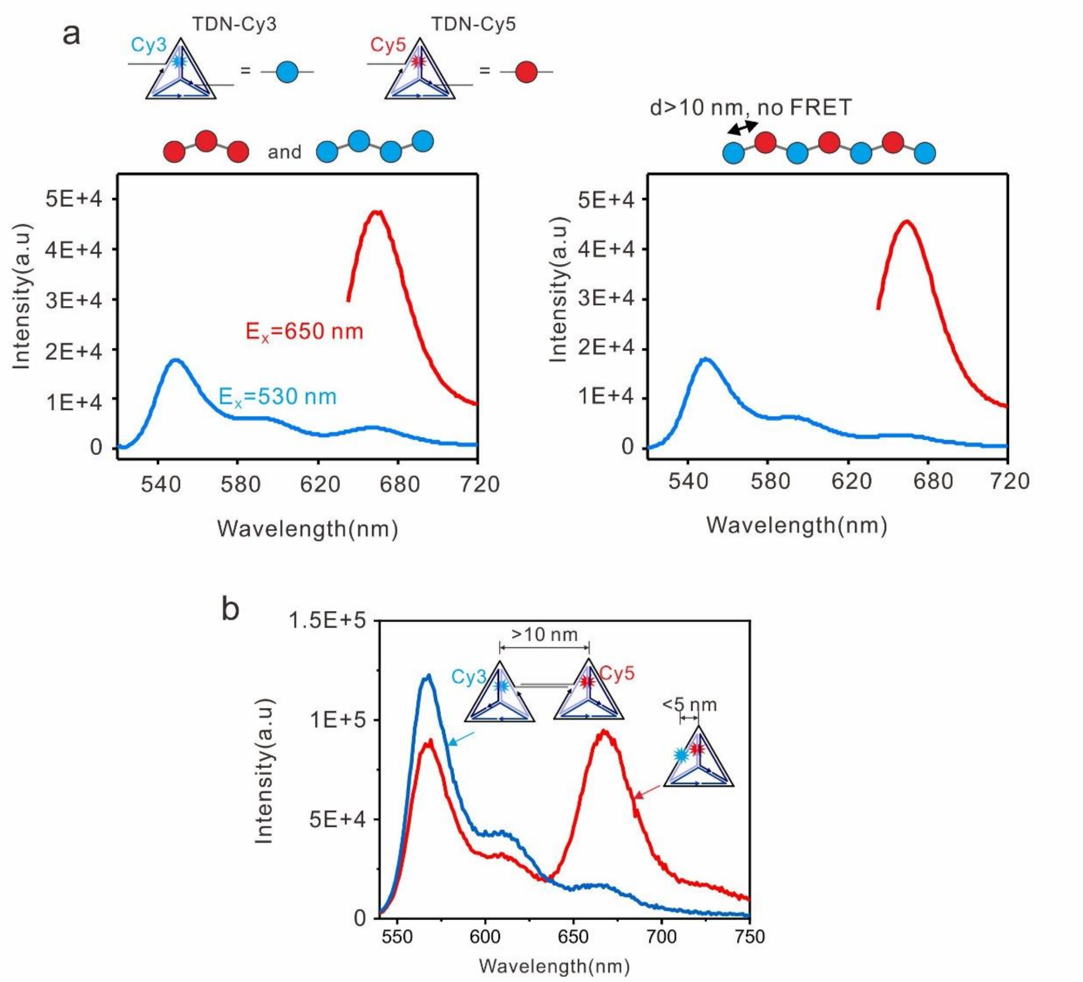

Supplementary Figure 10. Fluorescence spectra of FDFs with differently-coloured fluorophores. (a) Fluorescence spectra of the solution of a simple mixture of $[TDN\text{-}Cy3]_4$ and $[TDN\text{-}Cy5]_3$ and the solution of an $F_{2,3}$ structure carrying connected TDN-Cy3 and TDN-Cy5, suggesting that there is no obvious FRET between adjacent TDN nodes in the FDF structure. Blue, excitation (Ex) 530 nm; Red, Ex 650 nm. Concentration of each structure, 10 nM. (b) Fluorescence spectra of the solution of a TDN dimer [TDN-Cy3]·[TDN-Cy5] with Cy3 and Cy5 separately labeled on the two TDNs (blue), and a single TDN simultaneously labeled with Cy3 and Cy5 (red). Concentration of each structure, 50 nM. The latter structure shows apparent FRET effect (reduced emission of Cy3 and raised emission of Cy5, with Ex 514 nm).

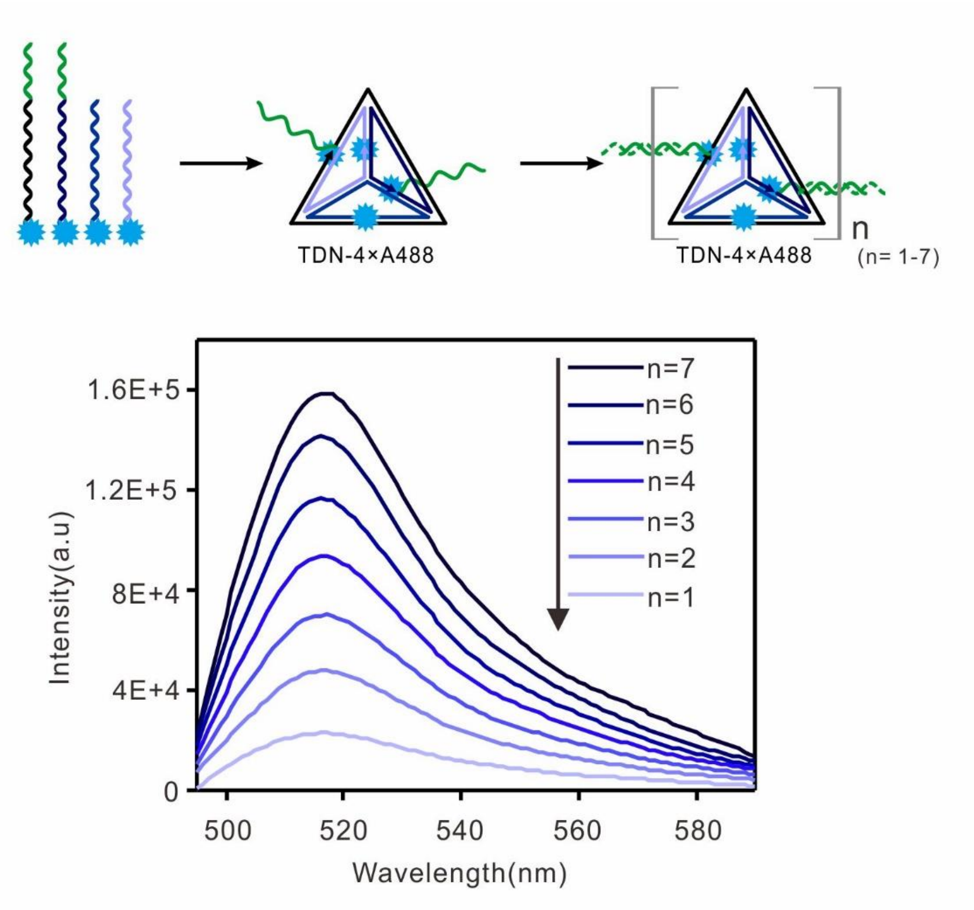

Supplementary Figure 11. Schematic and Fluorescence spectra of $F_{2,n}$ structures. n=1-7 with 4 A488 fluorophores on each TDN node. Ex, 488 nm. Concentration of each structure, 10 nM.

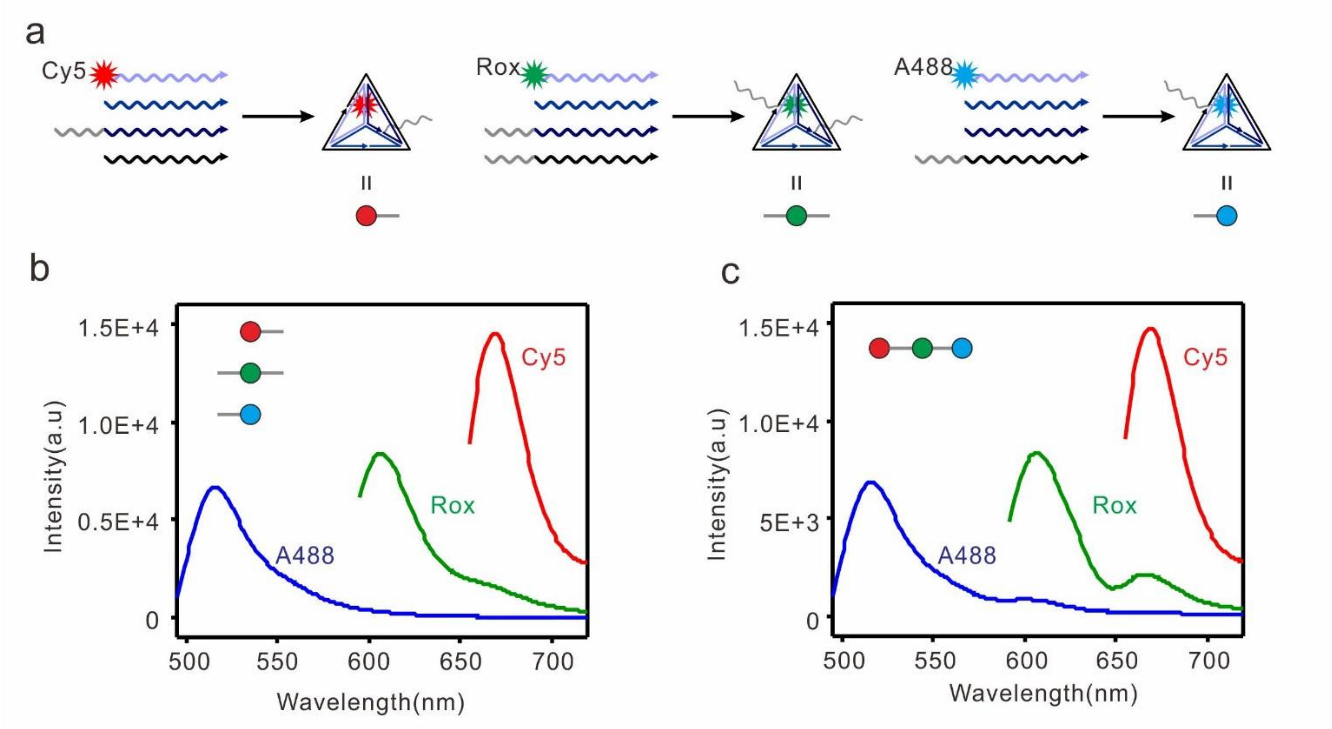

Supplementary Figure 12. Fluorescence spectra of differently-coloured TDN nodes and FDFs. a, Schematic illustration of TDN-Cy5, TDN-ROX and TDN-A488. b, Fluorescence spectra of the three coloured TDN nodes (Ex 650 nm, 588 nm and 488 nm, respectively at room temperature). c, Fluorescence spectra of the $F_{2,1}$ structure comprising TDN- Cy5, TDN-ROX, and TDN-488. Concentration of each structure, 10 nM.

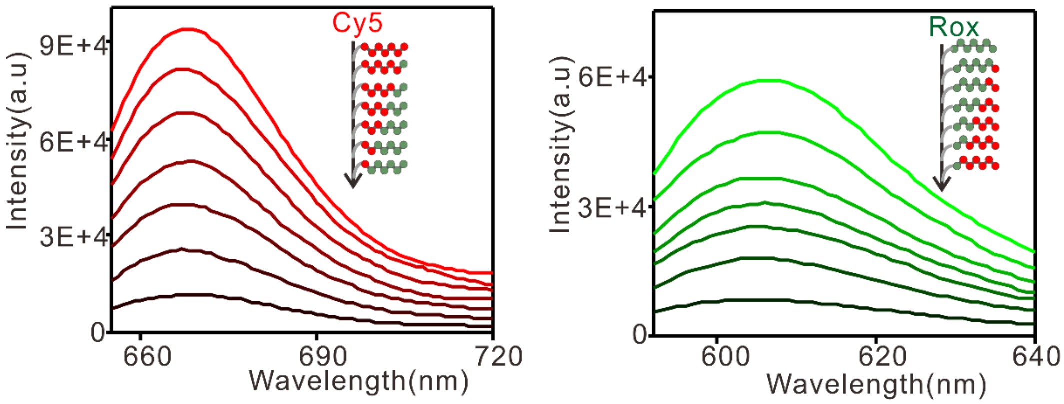

Supplementary Figure 13. Fluorescence spectra of Cy5 and ROX in FDF barcodes. Colour IDs: 700, 610, 520, 430, 340, 250, 160, and 070. Ex, 650 and 588 nm successively. Concentration of each barcode, 10 nM.

$F_{2,3}$

Cy5-Rox-A488	Spectra	R.Intensity Cy5	Rox	A488
700		7.2	0	0
610		6.4	1	0
520		5.2	2	0
430		4.0	3.1	0
340		3.0	4.0	0
250		2.2	5.3	0
160		1.0	6.4	0
070		0	7.4	0
061		0	6.4	1.0
052		0	5.0	2.0
043		0	4.3	3.2
034		0	3.5	4.2
025		0	2.4	5.0
016		0	1.2	6.2
007		0	0	7.0
106		1.0	0	6.2
205		2.2	0	5.3
304		3.1	0	4.3

Cy5-Rox-A488	Spectra	R.Intensity Cy5	Rox	A488
403		4.2	0	3.3
502		5.3	0	2.2
601		6.3	0	1.0
115		1.2	1.1	5.2
124		1.1	2.3	4.4
133		1.1	3.5	3.5
142		1.2	4.4	2.3
151		1.2	5.2	1.2
214		2.2	1.2	4.3
223		2.2	2.1	3.3
232		2.2	3.5	2.4
241		2.4	4.8	1.1
313		3.4	1.2	3.7
322		3.1	2.4	2.2
331		3.4	3.5	1.3
412		4.2	1.1	2.3
421		4.2	2.0	1.0
511		5.4	1.1	1.2

$F_{3,1}$

Cy5-Rox-A488	Spectra	R.Intensity Cy5	Rox	A488
400		4.2	0	0
310		3.0	1.0	0
220		2.0	2.0	0
130		1.1	3.1	0
040		0	4.2	0
031		0	3.1	1.1
022		0	2.3	2.4
031		0	1.0	3.1
004		0	0	4.2
103		1.0	0	3.2
202		2.1	0	2.1
301		3.0	0	1.0
112		1.1	1.1	2.1
121		1.1	2.3	1.3
211		2.0	1.1	1.0

$F_{4,1}$

Cy5-Rox-A488	Spectra	R.Intensity Cy5	Rox	A488
500		5.2	0	0
410		4.1	1.1	0
320		3.1	2.3	0
230		2.1	3.3	0
140		1.0	4.0	0
050		0	5.1	0
041		0	4.2	1.2
032		0	3.4	2.1
023		0	2.0	3.2
014		0	1.1	4.0
005		0	0	5.2
104		1.1	0	4.1
203		2.2	0	3.2
302		3.1	0	2.2
401		4.1	0	1.0
113		1.1	1.1	3.1
122		1.1	2.1	2.1
131		1.0	3.2	1.2
212		2.0	1.2	2.1
221		2.1	2.2	1.1
311		3.0	1.0	1.0

Supplementary Figure 14. FDF-encoded colour palette. Fluorescence spectra of each FDF structure can be regarded as the "fingerprint" of each FDF barcode. A488 channel, excitation (Ex) 488 nm, emission (Em) 495-590 nm; ROX channel, Ex 588 nm, Em 595-640 nm; Cy5 channel, Ex 650 nm, Em 655-720 nm. Fluorescence intensities of each channel were normalized to the intensity of one fluorophore in that channel. A three-digit ID is used to identify the barcodes. For example, "115" refers to a barcode containing 1 Cy5, 1 ROX, and 5 A488 fluorophores. All spectra were collected at 25 °C. Concentration of each barcode, 10 nM.

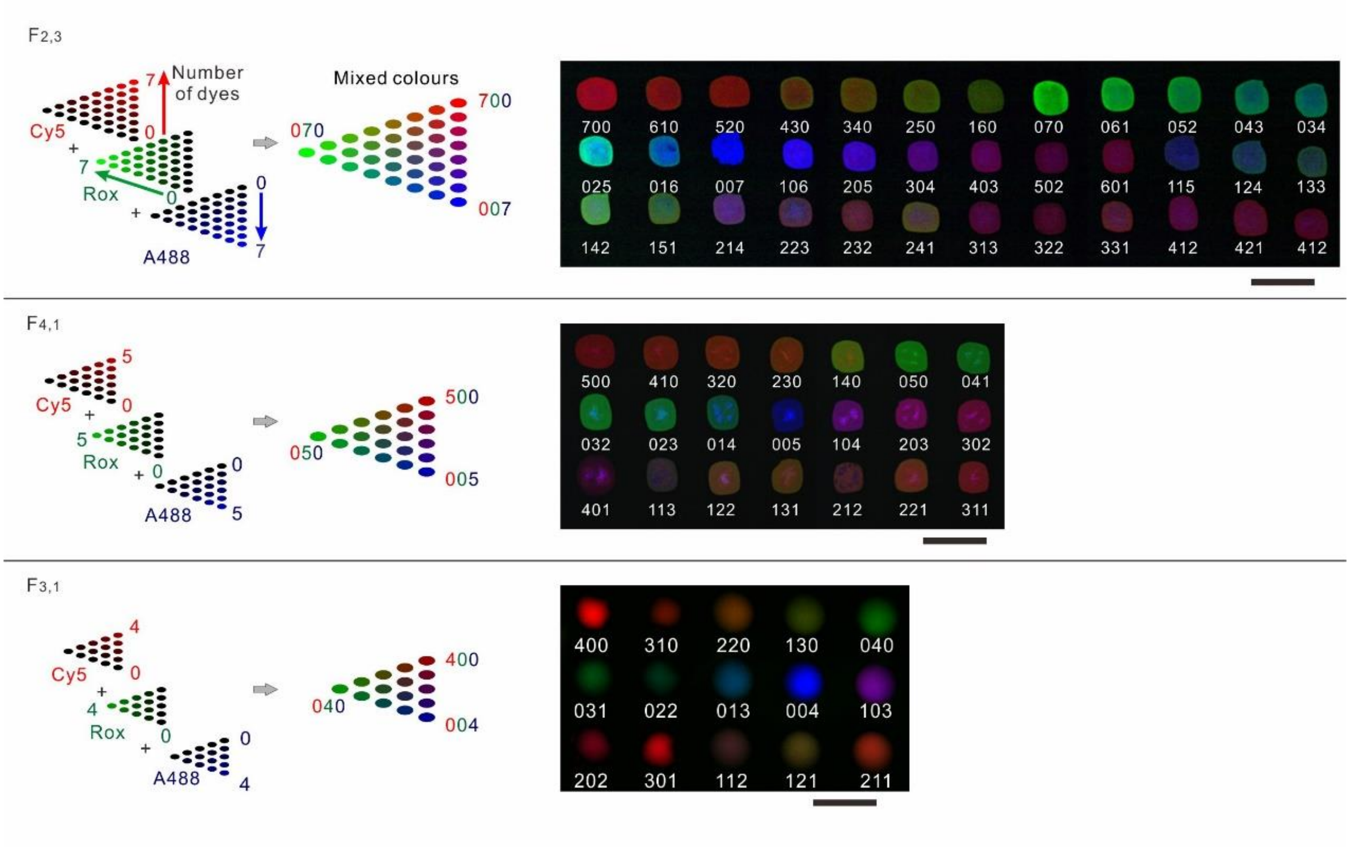

Supplementary Figure 15. Schematic and confocal images of the FDF-encoded colour palette. Red, Cy5 (Ex 633 nm, Em 650-720 nm); green, ROX (Ex 561 nm, Em 575-620 nm); blue, A488 (Ex 488nm, Em 495-550 nm). Confocal fluorescence images of FDF solution droplets are presented with pseudocolours. Scale bar, 250 µm. The three-digit colour IDs refer to the numbers of Cy5, ROX and A488 in a single FDF (e.g. 124 refers to the presence of 1 Cy5, 2 ROX and 4 A488).

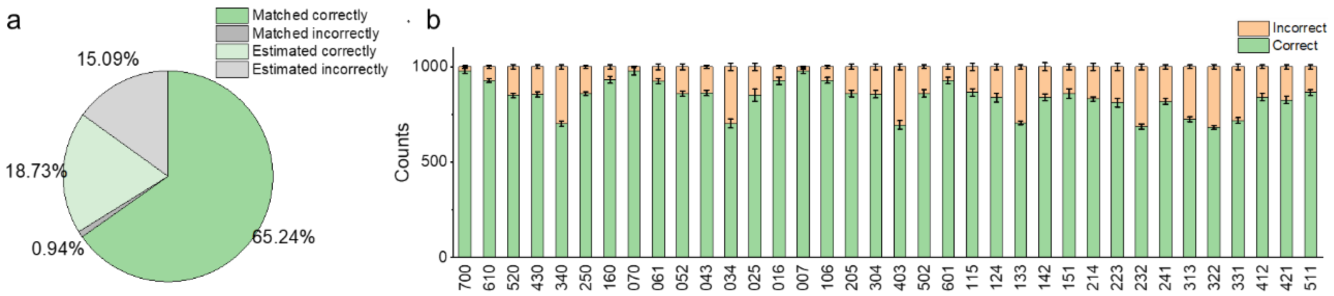

Supplementary Figure 16. Accuracy estimation of FDF barcode decoding. (a) Proportions of correct/incorrect decoding results from 36,000 computer-generated 7-node FDF barcode samples (36 barcode species, 1,000 samples per species) containing random errors in fluorophore counting (see Supplementary Methods). Among them, ~65% barcodes could correctly match the standard barcodes ("matched correctly"); ~1% matched to wrong standards ("matched incorrectly"); ~19% barcodes did not match to standards but could be correctly identified by Cosine Similarity analysis ("estimated correctly"); while the other ~15% barcodes obtained icorrect estimation answers ("estimated incorrectly"). Taken together, ~84% barcodes could be correctly decoded. (b) Decoding accuracy distribution of the 36 barcode species (N=1,000 each). Error bars represent standard deviations from 5 independent tests. Thus, we can conclude that the barcodes with higher fluorescence intensity ratios between different colours have higher accuracy in fluorescence decoding (e.g., barcode [6, 1, 0] or [1, 0, 6] is better than [3, 4, 0] or [3, 2, 2]).

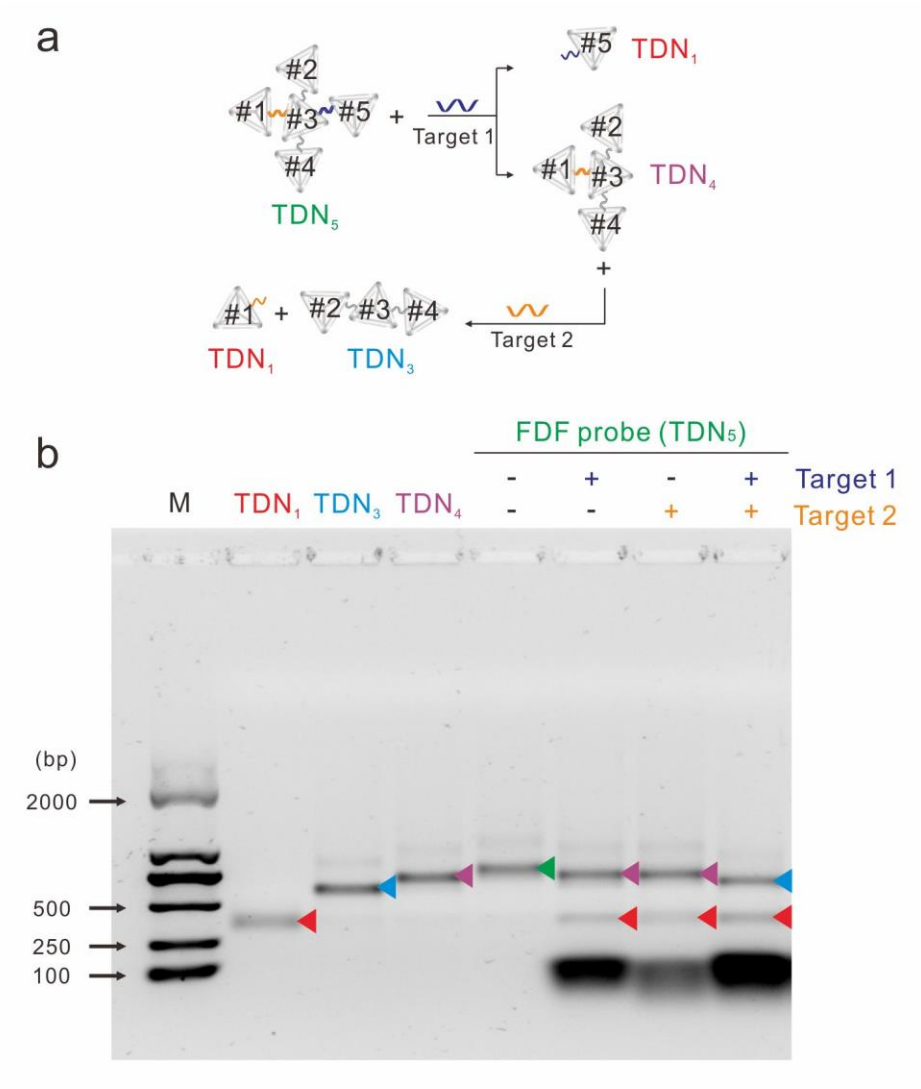

Supplementary Figure 17. Single-molecule recognition using FDF probes. (a) Schematic of single-molecule recognition experiment. Target 1 breaks the linkage between TDN #3 and #5 via strand displacement reaction (sequences listed in Supplementary Table S1). Target 2 breaks the linker between TDN #1 and #2. (b) Agarose gel electrophoresis of the structures in the single-molecule recognition reactions. Red, blue, purple, and green arrows marked the bands representing TDN_1, TDN_3, TDN_4, and the FDF probe (TDN_5), respectively. Thus, in response to (Target1+, Target2-) or (Target1-, Target2+,), the products contained TDN_1 (#1 or #5) and TDN_4. In response to (Target1+, Target2+), the products contained TDN_1 (#1 and #5) and TDN_3. DNA marker DL2000.

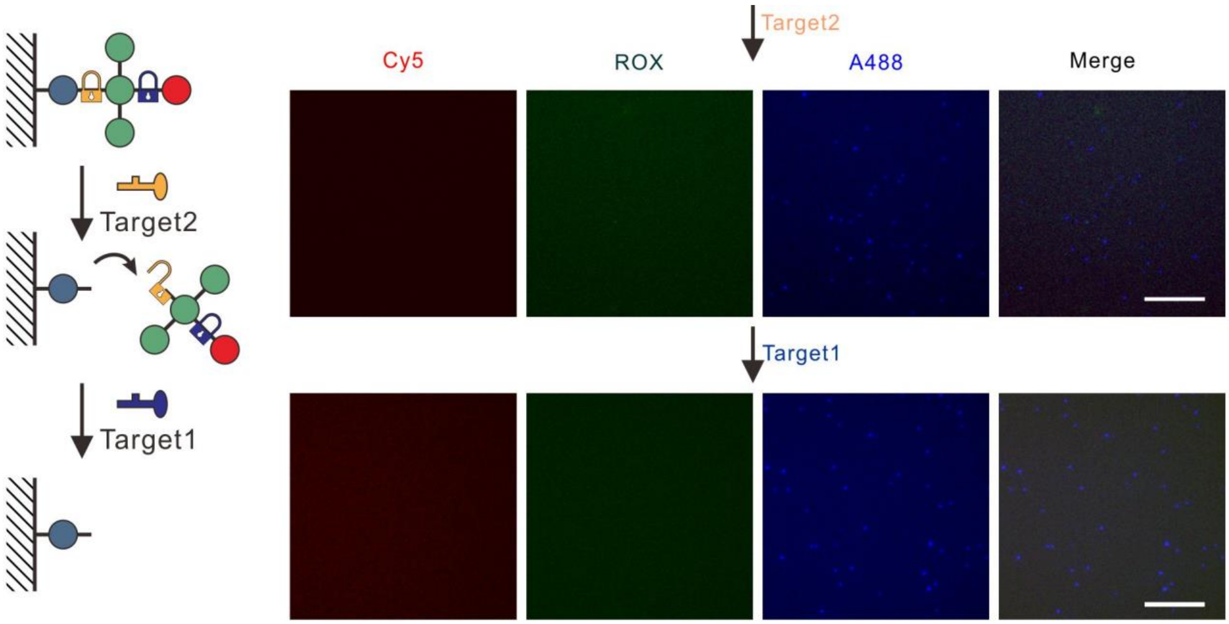

Supplementary Figure 18. TIRF images of the different sequence of the single-molecule detection. Scale bar, 10 μm.

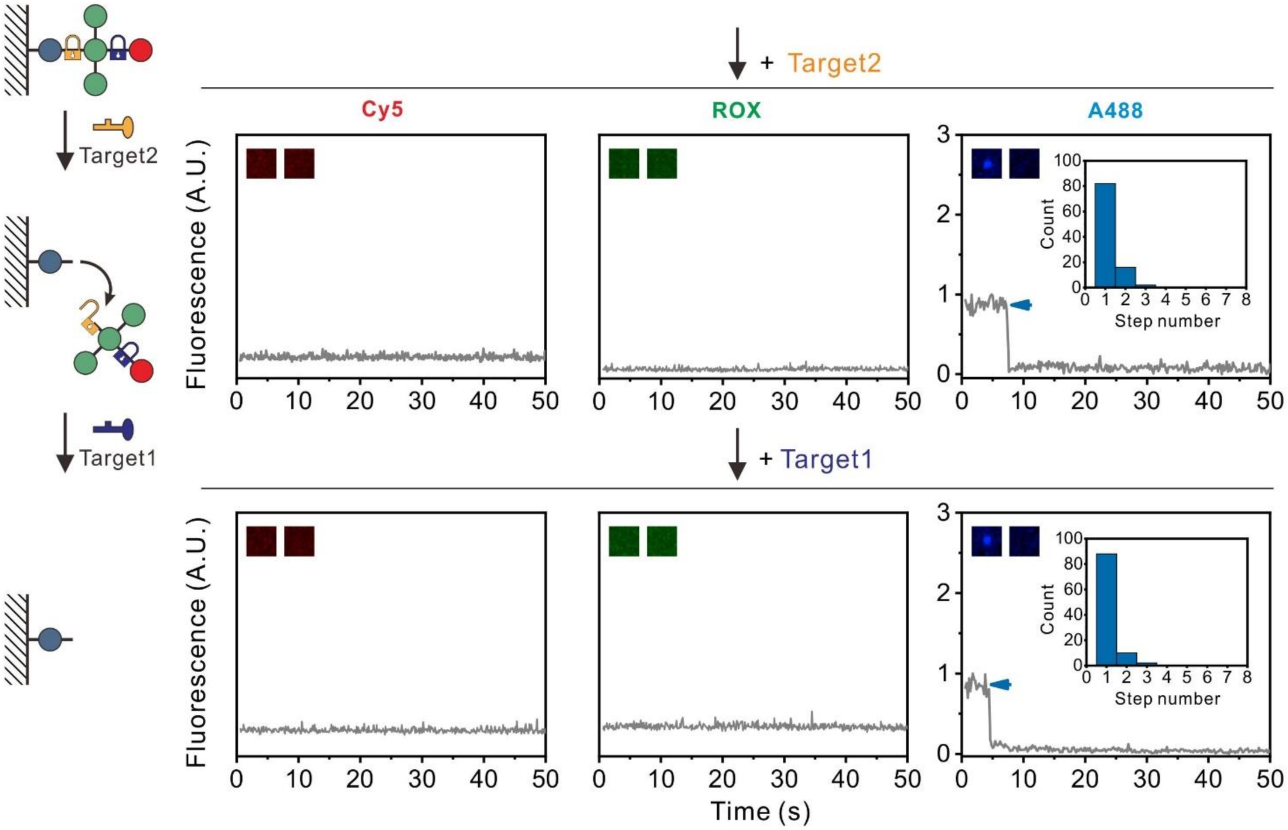

Supplementary Figure 19. Fluorescence quenching kinetics of a reverse input sequence of targets. N=100 each for the of quenching step counts.

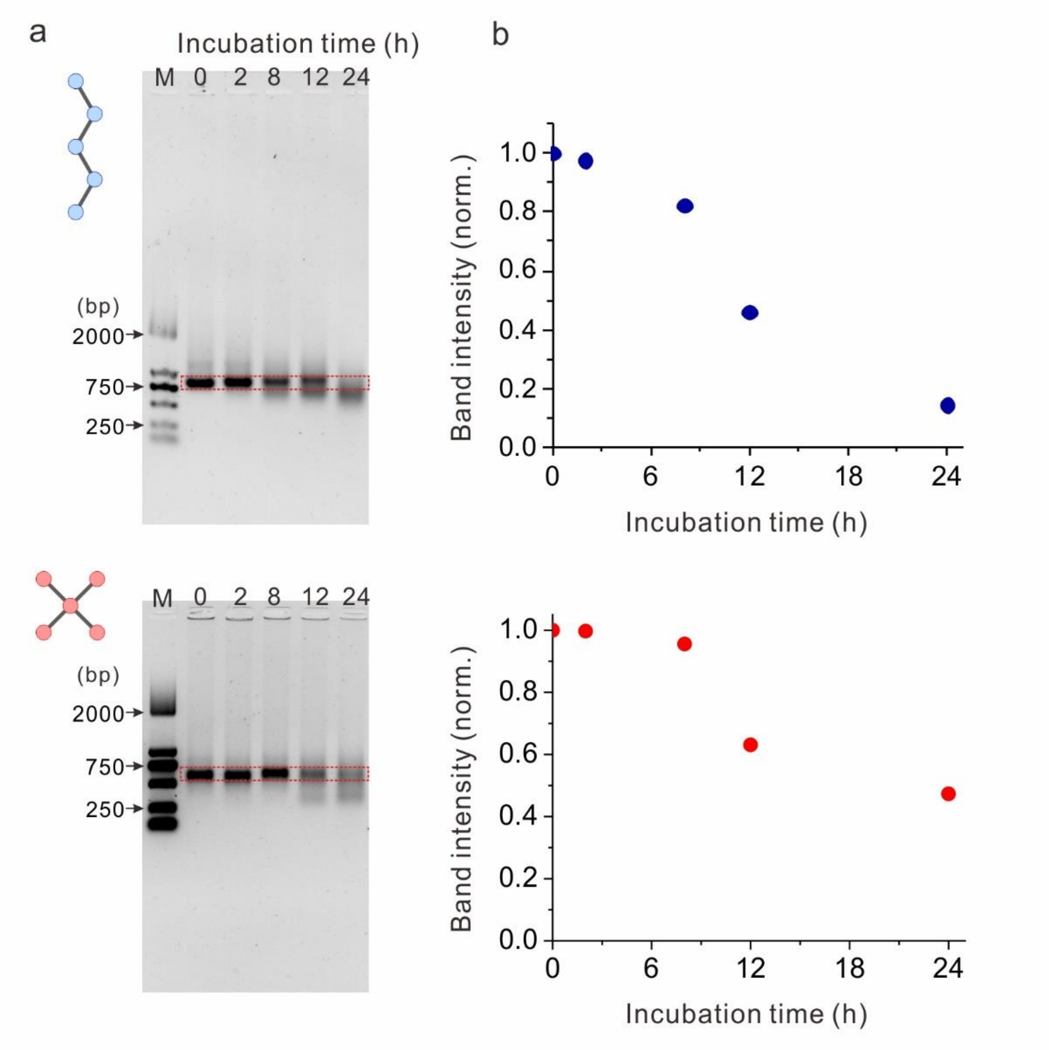

Supplementary Figure 20. Stability analysis of FDFs. (a) agarose gel images and (b) band intensity quantification (normalized to the intensity profile integral area of the lane, analyzed with ImageJ) of F2,2 and F3,1 separately incubated in 1640 medium (10% FBS) for 0 (control), 2, 8, 12 and 24 h.

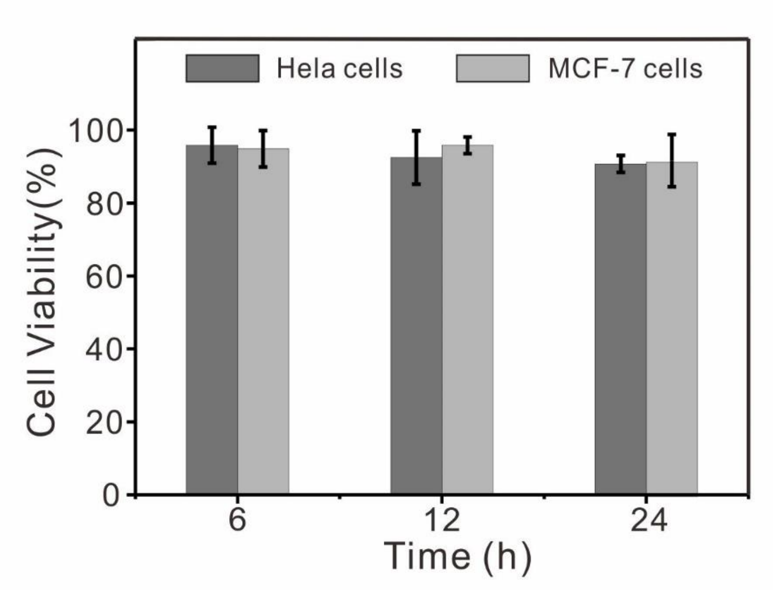

Supplementary Figure 21. MTT assay of HeLa and MCF7 cells incubated with FDF ($F_{4,1}$). Concentration of the FDF, 20 nM. Error bars represent s.d. from at least three independent tests.

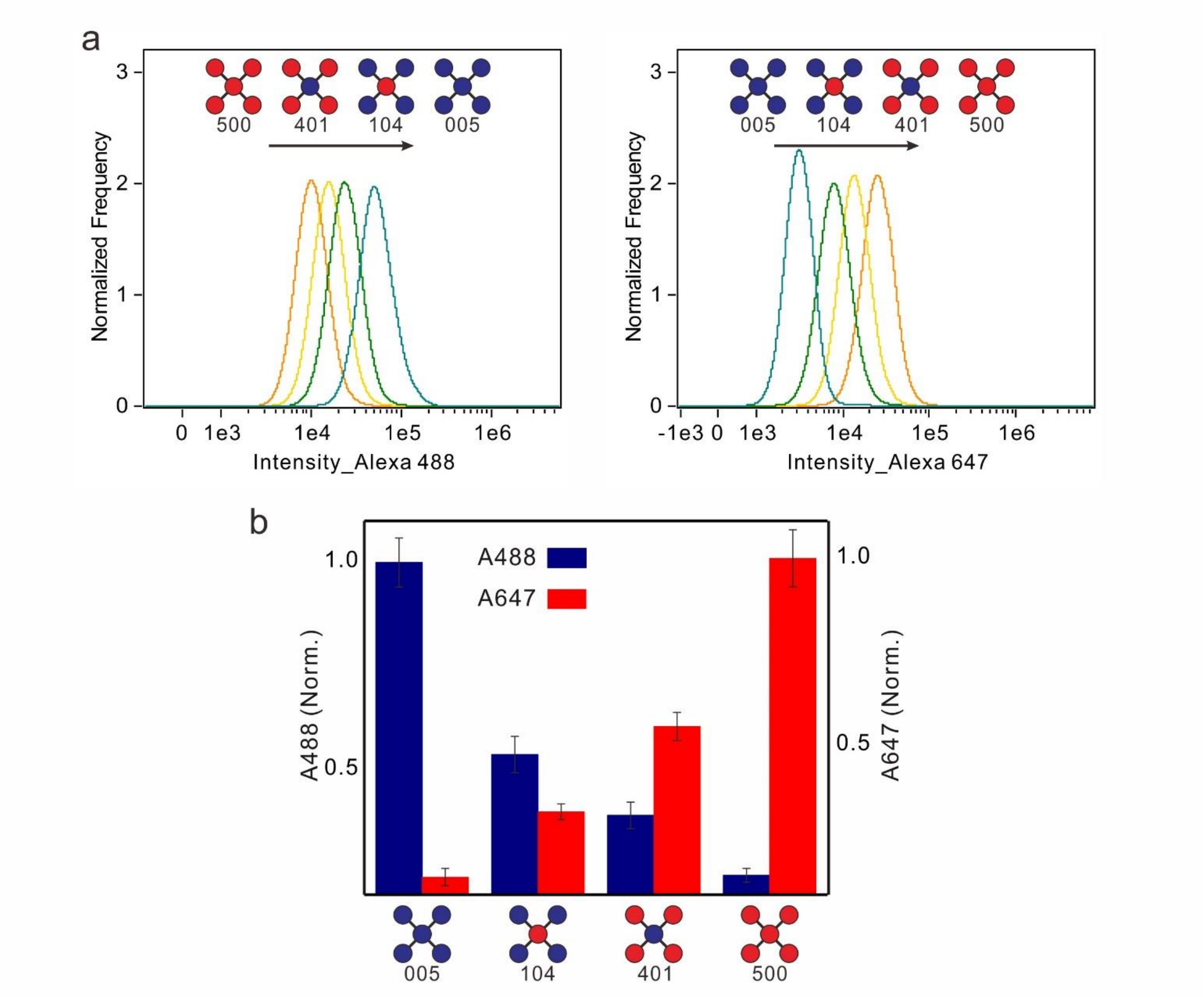

Supplementary Figure 22. Cytometric results of cells separately tagged with four $F_{4,1}$ barcodes. (a) Fluorescence intensity distributions of tagged cells in A488 channel and A647 channel, respectively. Colour IDs: 005, 104, 401, and 500. (b) Histograms of the mean fluorescence intensities of the four cell groups derived from (a). Error bars represent s.d. from three independent tests.

Supplementary References

1. Ouldridge, T.E., Louis, A.A. & Doye, J.P.K. Structural, mechanical, and thermodynamic properties of a coarse-grained DNA model. *J. Chem. Phys.* **134** (2011).
2. Doye, J.P.K. et al. Coarse-graining DNA for simulations of DNA nanotechnology. *Phys. Chem. Chem. Phys.* **15**, 20395-20414 (2013).
3. Russo, J., Tartaglia, P. & Sciortino, F. Reversible gels of patchy particles: Role of the valence. *J. Chem. Phys.* **131** (2009).
4. Lapham, J., Rife, J.P., Moore, P.B. & Crothers, D.M. Measurement of diffusion constants for nucleic acids by NMR. *J. Biomol. NMR* **10**, 255-262 (1997).
5. Wang, X. & Ha, T. Defining single molecular forces required to activate integrin and notch signaling. *Science* **340**, 991-994 (2013).
6. Li, Y., Cu, Y.T.H. & Luo, D. Multiplexed detection of pathogen DNA with DNA-based fluorescence nanobarcodes. *Nat. Biotechnol.* **23**, 885-889 (2005).
